# Transfer learning for versatile plant disease recognition with limited data

**DOI:** 10.3389/fpls.2022.1010981

**Published:** 2022-11-23

**Authors:** Mingle Xu, Sook Yoon, Yongchae Jeong, Dong Sun Park

**Affiliations:** ^1^ Department of Electronics Engineering, Jeonbuk National University, Jeonbuk, South Korea; ^2^ Core Research Institute of Intelligent Robots, Jeonbuk National University, Jeonbuk, South Korea; ^3^ Department of Computer Engineering, Mokpo National University, Jeonnam, South Korea

**Keywords:** plant disease recognition, transfer learning, vision transformer, self-supervised learning, few-shot learning, PlantCLEF2022

## Abstract

Deep learning has witnessed a significant improvement in recent years to recognize plant diseases by observing their corresponding images. To have a decent performance, current deep learning models tend to require a large-scale dataset. However, collecting a dataset is expensive and time-consuming. Hence, the limited data is one of the main challenges to getting the desired recognition accuracy. Although transfer learning is heavily discussed and verified as an effective and efficient method to mitigate the challenge, most proposed methods focus on one or two specific datasets. In this paper, we propose a novel transfer learning strategy to have a high performance for *versatile plant disease recognition*, on multiple plant disease datasets. Our transfer learning strategy differs from the current popular one due to the following factors. First, PlantCLEF2022, a large-scale dataset related to plants with 2,885,052 images and 80,000 classes, is utilized to pre-train a model. Second, we adopt a vision transformer (ViT) model, instead of a convolution neural network. Third, the ViT model undergoes transfer learning twice to save computations. Fourth, the model is first pre-trained in ImageNet with a self-supervised loss function and with a supervised loss function in PlantCLEF2022. We apply our method to 12 plant disease datasets and the experimental results suggest that our method surpasses the popular one by a clear margin for different dataset settings. Specifically, our proposed method achieves a mean testing accuracy of 86.29over the 12 datasets in a 20-shot case, 12.76 higher than the current state-of-the-art method’s accuracy of 73.53. Furthermore, our method outperforms other methods in one plant growth stage prediction and the one weed recognition dataset. To encourage the community and related applications, we have made public our codes and pre-trained model^^1^^.

## 1 Introduction

Keeping plants healthy is one of the essential challenges to having an expected and high yield. Traditionally, experts have to go to farms to check if plants are infected with diseases but deep learning enables the check to take place automatically based on their images. Because of the decent performance of deep learning, plant disease recognition has witnessed a significant improvement in recent years (Abade et al., 2021; Liu et al., 2021; Ngugi et al., 2021). To obtain a comparable recognition performance, a large-scale dataset is entailed to train a deep learning-based model. However, collecting images for plant disease is expensive and time-consuming. Besides, few images are normally available at the beginning of a plant disease recognition project when sanity checking should be executed before devoting more resources. Therefore, *limited dataset*, a situation where a few labeled images are accessible for some classes in the training process is one of the main issues in the literature (Fan et al., 2022). To facilitate this issue, many algorithms and strategies are proposed, such as data augmentation (Mohanty et al., 2016; Xu et al., 2022b; Olaniyi et al., 2022), transfer learning (Mohanty et al., 2016; Too et al., 2019; Chen J. et al., 2020; Xing and Lee, 2022; Zhao et al., 2022), few-shot learning (Afifi et al., 2020; Egusquiza et al., 2022), and semi-supervised learning (Li and Chao, 2021).

Although the challenge of a limited dataset is considered in many works, most of them merely focus on one or few specific datasets, such as the PlantVillage dataset (Mohanty et al., 2016; Too et al., 2019; Li and Chao, 2021), AI Challenger dataset (Zhao et al., 2022), tomato dataset (Xu et al., 2022b), wheat and rice dataset (Sethy et al., 2020; Rahman et al., 2020), cucumber (Wang et al., 2022), and apple leaf disease dataset (Fan et al., 2022). A basic question in this situation is whether a useful method for one dataset is helpful for other datasets. Further, there is a fundamental desire to find a robust method for most plant disease recognition applications. On the other hand, improving the application performance with a limited dataset is desired. For example, can we get a comparable result with only 20 training images for each class (20-shot)? To address these two issues, we propose a novel transfer learning strategy to achieve high performance for different limited datasets and various types of plants and diseases.

**Figure 1:**
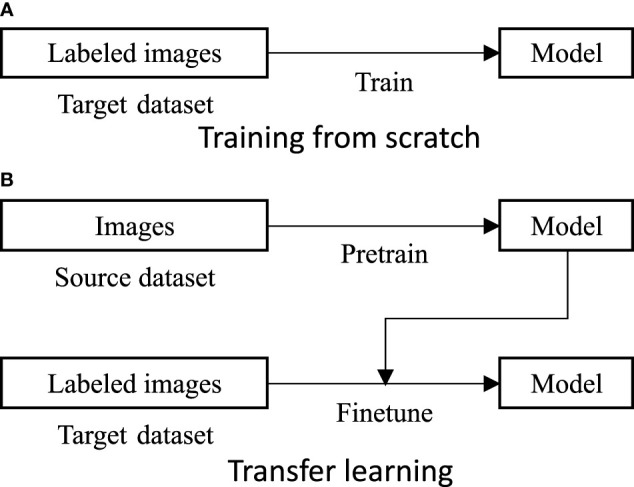
Training from scratch **(A)** and transfer learning **(B)**. Three key factors in transfer learning are the source dataset, the model, and the loss function to pre-train the model. These have all been undeveloped in plant disease recognition.

Via obtaining a good feature space, transfer learning aims to learn something beneficial for a target task with a target dataset from a source task with a source dataset (Pan and Yang, 2009). In plant disease recognition, a deep learning-based model is generally pre-trained in the source dataset and then fine-tuned in the labeled target dataset. As shown in **Figure 1**, it is understood that three key factors essentially lead to a positive transfer learning performance, a *desired source dataset*, *powerful model*, and suitable *loss function* to pre-train the model (Wu et al., 2018; Kornblith et al., 2019; Kolesnikov et al., 2020; Tripuraneni et al., 2020; He et al., 2022). However, the three factors have been undeveloped in plant disease recognition.

First, it is beneficial to have a *plant-related* dataset with a high number of images and classes (*large scale*), as well as *wide image variation*. For example, a plant-related source dataset could be better than the widely used ImageNet (Deng et al., 2009) for plant disease recognition, which has been verified (Kim et al., 2021; Zhao et al., 2022). Hence, finding a suitable source dataset is essential for plant disease recognition. Following this idea, PlantCLEF2022, a plant-related dataset with 2,885,052 images and 80,000 classes, was adopted for our paper.

Second, a model with higher performance in ImageNet or a source dataset may have a better performance in the target dataset with a transfer learning strategy (Kornblith et al., 2019). Convolution neural networks (CNN) (Krizhevsky et al., 2012; He et al., 2016) achieved the best accuracy for the ImageNet validation dataset. Simultaneously, the attention mechanism has been leveraged to boost the performance of plant disease recognition (Yang et al., 2020; Qian et al., 2022; Zhao et al., 2022). In recent years, Vision Transformer (ViT) (Dosovitskiy et al., 2020), a general model of attention mechanism, has become a hot topic in the computer vision community and outperforms CNN-based models. For example, MAE (He et al., 2022) scores 85.9 inaccuracy for the ViT-L model which is higher than Resnet50 and ResNet152 with scores of 79.26 and 80.62, respectively. Therefore, for plant recognition, ViT-based models with a transfer learning strategy are promising but still underdeveloped (Wang et al., 2022).

Third, the supervised loss function inevitably pushes the model to learn source task-related features that may not be helpful for the target task (Wu et al., 2018). In contrast, the self-supervised loss function eases the issue by introducing a pretext task, such as contrast loss (Wu et al., 2018) and reconstruction loss (He et al., 2022). Thus, a ViT mode pre-trained in the PlantCLEF2022 dataset with a self-supervised loss function is assumed to be better than the current popular transfer learning strategy that is pre-trained on a CNN-based model in the ImageNet dataset with a supervised loss function (Mohanty et al., 2016; Yang et al., 2020; Abbas et al., 2021; Fan et al., 2022; Yadav et al., 2022).

Besides, the transfer learning strategy is slightly problematic when considering computing devices and the large-scale PlantCLEF2022 dataset. To be more specific, training a ViT model 800 epochs in PlantCLEF2022 as MAE (He et al., 2022) requires more than five months with four RTX 3090 GPUs. To reduce the computing cost, we utilize a dual transfer learning strategy, where a public ViT model pre-trained in ImageNet with a self-supervised loss function is trained in the PlantCLEF2022 dataset with a supervised loss function. In this way, we only spend about 15 days training the model in PlantCLEF2022. We emphasize that our dual transfer learning is different from (Azizi et al., 2021; Zhao et al., 2022) due to the following facts, aiming to reduce the cost of pre-training a model, large-scale PlantCLEF2022 dataset, and employing a ViT-based model.

To summarize, our paper will make the following contributions:

- We propose a novel transfer learning to achieve versatile plant disease recognition with a plant-related source dataset PlantCLEF2022, ViT model, and self-supervised learning to pre-train the model.
- We utilize dual transfer learning to save computation costs, considering the large-scale PlantCLEF2022 dataset.
- We validate our method in 12 plant disease datasets and our method surpasses the current widely used strategy by a large margin. Specifically, we score an average testing accuracy of 86.29 in a 20-shot case, 12.76 higher than the widely used strategy.
- Our transfer learning strategy also outperforms other methods in one plant growth stage prediction and one plant weed recognition, which suggests that our strategy contributes beyond plant disease recognition.

## 2 Material and method

### 2.1 Plant disease datasets

To validate the generalization of transfer learning and deep learning, we executed our method in fourteen public datasets, thirteen related to plant disease recognition. To be more specific, we used PlantVillage (Hughes et al., 2015), PlantDocCls (Singh et al., 2020), Cassava (Ramcharan et al., 2017), Apple2020 (Thapa et al., 2020), Apple2021 (Thapa et al., 2021), Rice1426 (Rahman et al., 2020), Rice5932 (Sethy et al., 2020), TaiwanTomato^2^, IVADLTomato and IVADLRose^3^, CitrusLeaf (Rauf et al., 2019), CGIARWheat^4^, and PDD271* (Liu et al., 2021). More details of the datasets are shown in **Table 1** while three random images for each class are displayed here^^5^^.

**Table 1:** Information of the used plant disease recognition datasets.

Dataset	Images	Classes	Highlights
PlantVillage	54,305	38	Covers 14 types of plants. Each image is taken in controlled conditions and only includes one leaf in the center. Some diseases are spilt into two cases according to their severities, early and late. Each class has more than 273 images. All images are the same height and width, 256*256.
PlantDocCls	2,576	27	Includes 13 plants. The images are collected from the Internet with diverse heights and widths and most of the images are taken in real field conditions. The original training and testing dataset include 2,340 and 236 images, respectively.
Cassava	21,397	5	The images are taken in real field conditions and thus have wide variations, such as background, illumination, and leaf scales. All images have the same height and width, 800*600.
Apple2020	3,642	4	Taken in real field conditions. One leaf may include more than one type of disease and those images are labeled as one class. All images are the same size, 2048*1365.
Apple2021	18,632	6	An updated version of Apple2020 but with 2 more classes. All images are the same size, 4000*2672.
Rice1426	1,426	9	Images are taken in both real filed and controlled conditions. The images are not just related to leaves, but also other organs, stems, and grains. Images are in 224*224 resolution.
Rice5932	5,932	4	Only includes rice leaf images with different scales. All images are resized to 300*300.
TaiwanTomato	622	5	One image may include one or multiple leaves taken in either controlled conditions or real field conditions. There are 495 and 127 images in the original training and testing dataset, respectively. All images are resized to 227*227.
IVADLTomato	3,021	9	The original dataset includes more images in an unbalanced way. We limited the number for each class to less than 520. The original images have a large height and width, and we resized the images to 520*520 to save disk space.
IVADLRose	3,132	6	Similar to IVADLTomato, we limited the number for each class and resized the images.
CitrusLeaf	609	5	Images are taken in controlled conditions and resized to 256*256. We only used the leaf parts from the original Citrus dataset.
CGIARWheat	876	3	Includes leaves, stems, and whole plants. Images are taken from different viewpoints with diverse distances and different image sizes.
PDD271*	2,710	271	Covers fruit trees, vegetables, and field crops, with huge image variations. Ten images for each class are available as samples.

**Figure 2:**
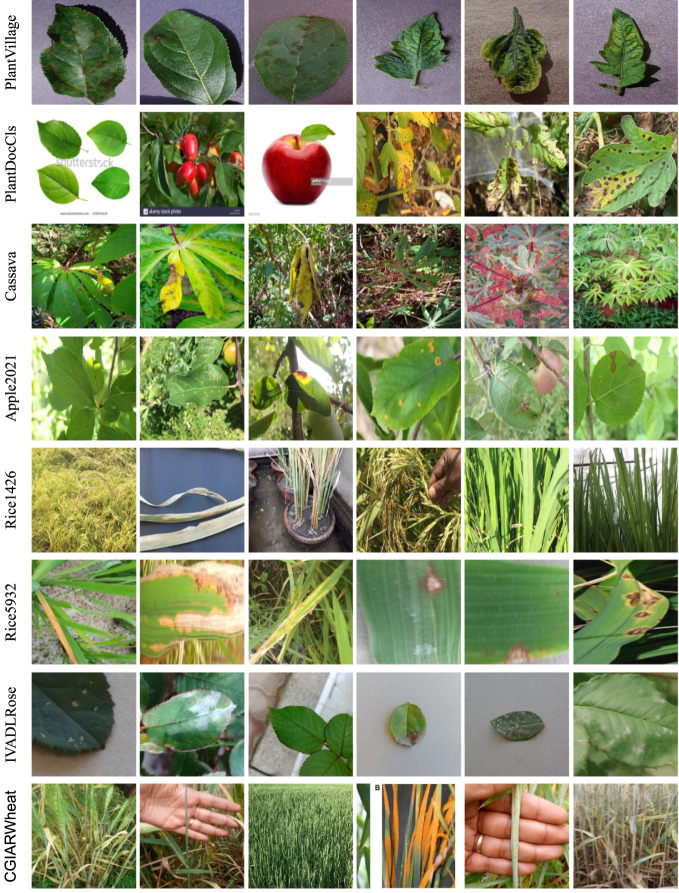
Image examples from different datasets. We recognize that there are image variations [40], such as background, the shape of leaves, illumination, and scale.

The datasets are considered from several viewpoints. **Figure 2** gives a glance at some images in the datasets. First is the *number of images and the number of classes*. Generally, the more classes and fewer images, the more difficult the recognition task. PDD271 covers 271 classes, including fruit trees, vegetables, and field crops, but unfortunately, it is not public. Only ten samples for each class are available and therefore, we adopted it as a few-shot learning task. In contrast, most of the public datasets only involved one type of plant, such as rice (Rahman et al., 2020; Sethy et al., 2020) or apple (Thapa et al., 2020; Thapa et al., 2021). Besides, the number distribution of classes may cause class-imbalance trouble, in which the trained model may have higher performance for the class with a dominant number of images in the training stage. Second, *the conditions the images were taken in* matters since controlling the conditions reduces the variation in the collected images, such as background and illuminations. A previous work (Barbedo, 2019) proves that controlling the conditions or masking the background out can improve recognition performance. Third, the *organs* of plants in images are also important. The main organs in the datasets are leaves, but also include some fruits, stems, and whole plants. Interestingly, different leaves of plants have heterogeneous shapes that may result in various performances with the same model. For example, the leaves of cassava are far different from their counterparts in apple and tomato plants. Especially, some images in PDD271 are captured with part of a leaf, not the whole leaf as in PlantVillage. Fourth, the *scale* of the images is also essential to the performance. The scale is related to the distance between the camera and the plant when taking pictures. For example, the leaves in PlantVillage and Apple2020 have a similar scale while the images in Rice1426 are on different scales. Fifth, *image size*,i.e. height and width, may incur challenges for recognition tasks as the disease phenomenon may not be clear enough in small-size images. To summarize, we emphasize that image variations (Xu et al., 2022a) in the dataset have an influence on training models and their corresponding performance, and thus, recognizing the image variations is significant to understanding the dataset.

### 2.2 PlantCLEF2022 dataset

**Figure 3:**
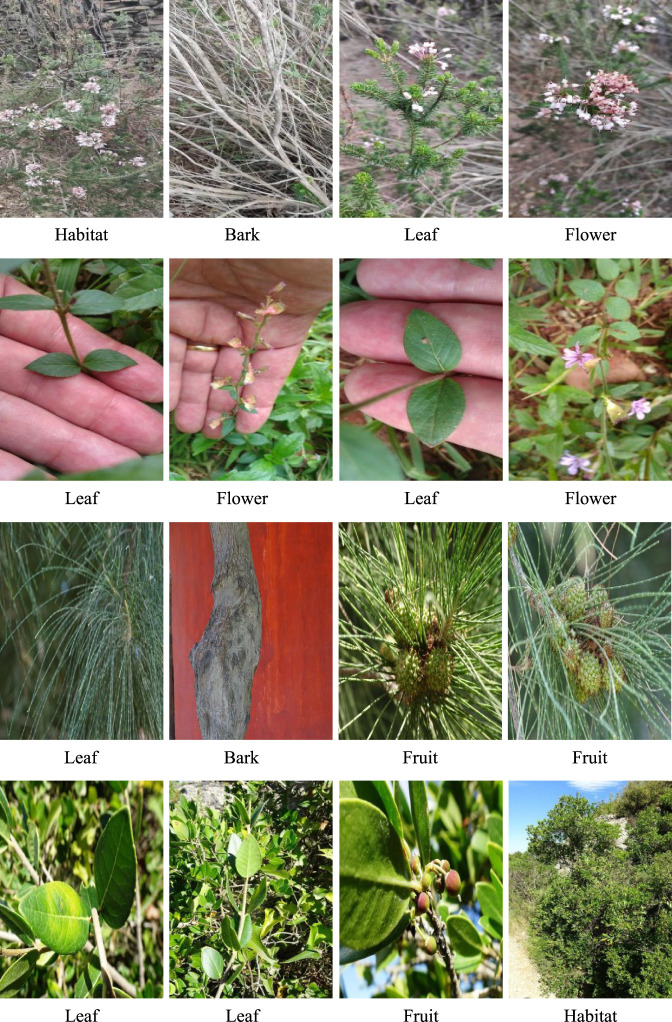
Different interests or organs in PlantCLEF2022 testing dataset.

**Figure 4:**
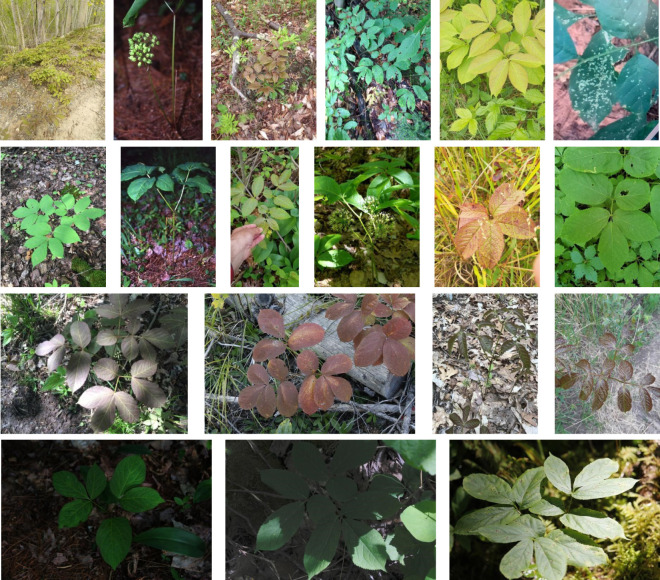
Images of Aralia Nudicaulis L. species from PlantCLEF2022 dataset. The images from the same plant species are heterogeneous in the background, illumination, color, scale, etc.

PlantCLEF2022^6^ was originally a challenge to identify the plant species based on their images. The trusted training dataset, PlantCLEF2022, annotated by human experts with 2,885,052 images and 80,000 classes, is leveraged and used as the default PlantCLEF2022 dataset in this paper. Each class in the dataset is limited to no more than 100 images and has 36.1 images on average. As shown in **Figure 3**, the images cover plant habitat (environment or background) and organs such as the leaf, fruit, bark, or stem. Essentially, plants can be recognized based on multiple pieces of visual evidence, instead of only one piece of evidence (Xu et al., 2022c). Besides, the images belonging to one class embrace huge variations. As displayed in **Figure 4**, the variations include background, illumination, color, scale, and image size.

**Why PlantCLEF2022?** We recognize that three characteristics make PlantCLEF2022 beneficial to plant disease recognition with transfer learning strategy, i.e., *plant-related, large-scale, and wide variations*. First, it is accepted that a large-scale related source dataset contributes to the target task. As the PlantCLEF2022 dataset is plant-related and on a large scale, even when compared to ImageNet (Deng et al., 2009), it can be beneficial to plant disease recognition and related tasks, such as growth stage prediction. Second, the PlantCLEF2022 dataset has wide variations as mentioned before, by which we can learn a better feature space when using it to pre-train a model. Arguably, the variations in PlantCLEF2022 are much stronger than all of the plant disease datasets introduced in Section 2.1. We have noticed that finding this kind of dataset for plant disease cognition tasks is one of the main interests in recent years. In the beginning, ImageNet made a significant contribution as a source dataset. Recently, the AI Challenger dataset, a little bit bigger than PlantVillage but with small variations as most of the images are taken in controlled conditions, is considered as a source dataset (Zhao et al., 2022). Although it is plant-related, the AI Challenger dataset is far behind when compared to PlantCLEF2022 because of its number of images and classes and poor image variations.

### 2.3 Dual transfer learning

**Figure 5:**
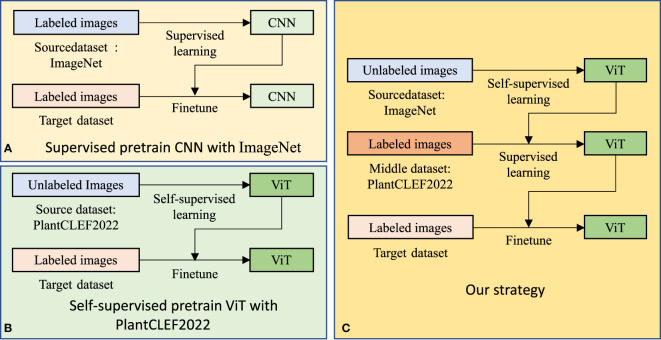
Transfer learning strategies for plant disease recognition. Our strategy differs from the current popular transfer learning strategy **(A)** in the source dataset, model, and loss function. Furthermore, we adopt dual transfer learning **(C)** to save computation time by utilizing the public pre-trained model, compared to **(B)**.

To achieve versatile plant disease recognition with a limited dataset, we believe that, under the transfer learning paradigm, a large-scale related dataset, PlantCLEF2022, and a powerful model are beneficial. Hence, we designed a dual transfer learning model, taking the computation load and device into consideration. As shown in **Figures 5A, C**, our transfer learning consists of three steps with transfer learning occurring twice.

**Figure 6:**
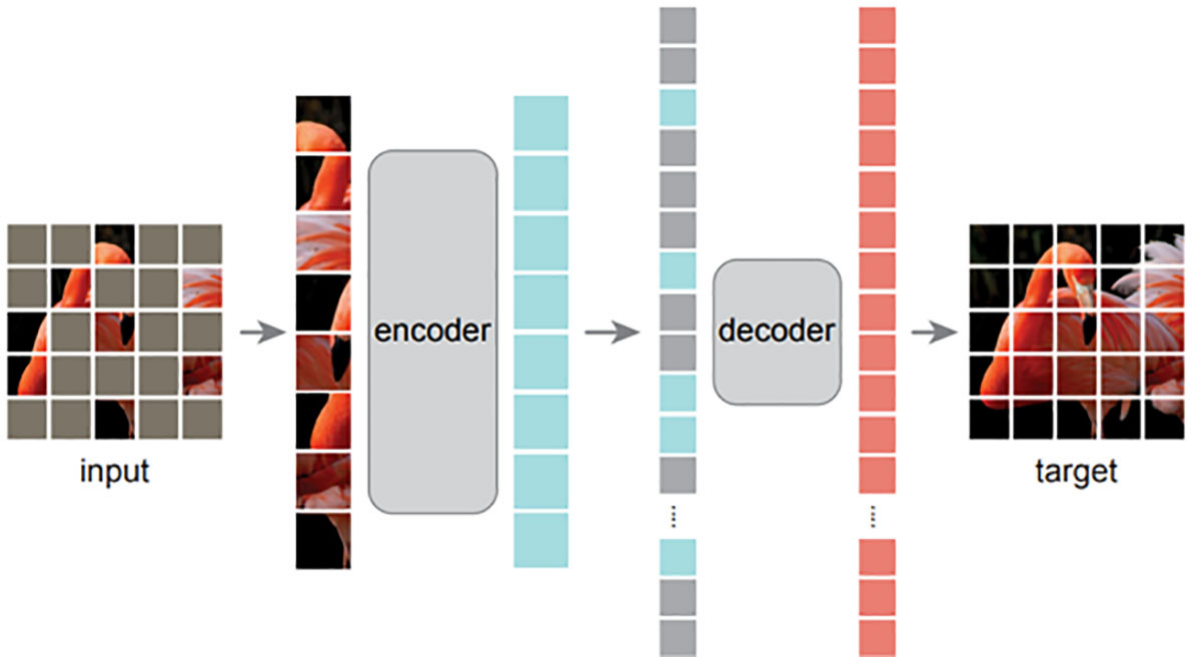
The high-level architecture of MAE [13]. With MAE, an image is split into patches that are then randomly blocked. The unblocked patches are fed to an encoder, followed by a decoder to reconstruct the whole input image. After the unsupervised pre-training, the decoder is discarded and only the encoder is utilized in the downstream task. The input is not blocked and a specific classifier is added after the encoder when fine-tuning the model in a target task.

In the first step, a vision transformer (ViT) model is pre-trained with the ImageNet (Deng et al., 2009) in a self-supervised manner, reconstruction loss. We emphasize here that we directly adopted the pre-trained model from masked autoencoder (MAE) (He et al., 2022), instead of training the model ourselves. Simultaneously, we argue that superior pre-trained models are essential for better plant disease recognition, even if the models have the same architecture. The experiments in the following section prove that the original pre-trained ViT model (Dosovitskiy et al., 2020) performs worse than MAE (He et al., 2022). As shown in **Figure 6**, MAE is a composite of an encoder and a decoder that are optimized by a reconstruction loss, *ℒ*_*recon*_=||*input*, *target*||_2_ where *input* is the original image and *target* denotes the reconstructed image. During the training process, the original image *input* is split into several patches that are randomly blocked. The encoder aims to extract necessary information from the blocked image and the decoder is required to fill the blocked patches. As the optimization does not require labels, it falls under self-supervised learning.

The decoder in MAE is discarded and the encoder is utilized in the second step, followed by a linear layer and a softmax operation to do classification. The encoder and the added linear layer are fine-tuned in the PlantCLEF2022 dataset, optimized by the cross entropy loss, *ℒ*_*ce*_=−*log*(*p*(*y*_*j*_)) where *j* is the ground truth index and *p*(*y*) is the output of softmax operation. Different from the first step, the input is not split into patches and blocked. The main characteristic of the second step is the PlantCLEF2022 dataset, related to the plant disease recognition dataset. We highlight that the second step is outlined and trained in our previous paper (Xu et al., 2022c) for the PlantCLEF2022 challenge and thus is not outlined and trained in this paper.

In the third step, the added linear layer in the second step is replaced by a new linear layer. To be clear, the encoder and the new linear layer in this step are fine-tuned in a specific plant disease recognition dataset. The cross-entropy loss is again utilized to optimize the whole network. As mentioned before, the first and second steps are executed in other papers and thus only the third step is required for this paper. We have termed our strategy dual transfer learning since the model is trained with two other datasets and transferred twice.

We believe that the first step is not mandatory for better performance in versatile plant disease recognition but contributes to the reduction of the training time for the whole system. As shown in **Figure 5B**, we can pre-train a model in the PlantCLEF2022 dataset and then fine-tune it for the plant disease dataset. Unfortunately, this setting may entail a long training epoch in PlantCLEF2022 to have a better performance, such as 800 epochs in MAE (He et al., 2022). In contrast, we only train 100 epochs for the second step and hence can save time. Besides, by training an MAE model in a self-supervised way, one decoder is trained at the same time which needs more time for one epoch. Therefore, our dual transfer learning reduces training time *via* utilizing the public model from MAE (He et al., 2022).

## 3 Experiment

### 3.1 Experimental settings

**Dataset.** For each original dataset in **Table 1**, we split them into training, validation, and testing datasets. The training dataset is leveraged to train the models while the validation one is only used to choose the best-trained model from different epochs. Then, the best model is evaluated in the testing dataset. If there is a testing dataset with annotations in the original dataset, we directly used the original testing dataset. Otherwise, the whole original dataset is split into training, testing, and validation datasets in different percentages or an exact number of images. To be more specific, the original testing datasets in PlantDocCls and TaiwanTomato are directly used while a new testing dataset is made for other datasets.

For each plant disease dataset, we consider two training cases, generic and few-shot cases. Different percentages of the training dataset are utilized in the generic case, such as 20% and 40%, while only several images for each class are taken to train the model in the few-shot case. To summarize, we set eight dataset modes, as shown in **Table 2**, four percentages as training in generic cases and 4 types of few-shot cases. Except for ratio80, 20% is taken for the validation and testing datasets for all experiments. The validation and testing datasets are the same for the generic and few-shot cases. Furthermore, the dataset splitting was randomly executed once only, by which the images of each dataset mode are fixed for all compared models or strategies. Although the percentage of validation and testing datasets is the same for most of the dataset modes, the images are different because of a different random process.

**Table 2:** The settings in different dataset modes for the original dataset without labeled testing dataset.

Dataset case	Dataset mode	Training	Validation	Testing
**Generic case**	Ratio20 Ratio40 Ratio60 Ratio80	20% 40% 60% 80%	20% 20% 20% 10%	20% 20% 20% 10%
**Few-shot case**	1-shot 5-shot 10-shot 20-shot	1 5 10 20	20% 20% 20% 20%	20% 20% 20% 20%

The splitting was random once only, by which the images of each dataset mode are fixed for all compared models or transfer learning strategies. Although the percentage of validation and testing dataset was the same for most of the dataset modes, the images are different because of a different random process.

**Comparison methods**. To validate our method, we designed several comparisons with different strategies or models. To choose the compared methods, we held to the following features: with transfer learning or without transfer learning, CNN-based or ViT-based, supervised or self-supervised, and trained with PlantCLEF2022 or not. Simultaneously, we do not want to pre-train the models because of our lack of GPUs and the almost 3 million images in PlantCLEF2022. Based on these two ideas, the compared methods are described below and more interesting methods are listed in **Table 3** with their corresponding characteristics.

- RN50. A ResNet50 model is trained from scratch with the target datasets shown in **Table 1**.
- RN50-IN. A ResNet50 model is pre-trained with the ImageNet (IN) dataset in a supervised way and then fine-tuned in the target datasets.
- MoCo-v2. A MoCo-v2 model is pre-trained with the ImageNet dataset in a self-supervised way and then fine-tuned in the target datasets.
- ViT. A ViT-large (Dosovitskiy et al., 2020) model is trained from scratch with the target datasets.
- ViT-IN. A ViT-large model is pre-trained with the Imagenet dataset in a supervised way and then fine-tuned in the target datasets.
- MAE. A ViT-large model is pre-trained with the ImageNet dataset in a self-supervised way. Specifically, MAE (He et al., 2022) uses reconstruction loss to learn better performance with a high occlusion.
- Our model. We fine-tuned a ViT model from MAE with the PlantCLEF2022 dataset and then fine-tuned it again with the target datasets.

**Table 3:** The characteristics of the compared methods.

Case	Name	Model	ImageNet	PlantCLEF2022
1 2 3 4 5 6 7 8 9 10	RN50 RN50-IN - MoCo-v2 - ViT ViT-IN - MAE Ours	CNN CNN CNN CNN CNN ViT ViT ViT ViT ViT	N/A Supervised N/A Self-supervised Self-supervised N/A Supervised N/A Self-supervised Self-supervised	N/A N/A Self-supervised N/A Supervised N/A N/A Self-supervised N/A Supervised

N/A denotes not available or not used. We evaluated the compared methods from these viewpoints: no pre-training process because of our lack of GPUs, and showing the impacts of the basic model (CNN orViT), supervised or self-supervised, plant-related dataset (ImageNet or PlantCLEF2022), and dual transfer learning strategy. The named methods are compared in our paper while the other methods are encouraged and left for future studies considering the availability of GPUs.

We noticed that there were several other possible strategies. For instance, it is interesting to directly pre-train a ViT model with only the PlantCLEF2022 dataset in a self-supervised manner, no ImageNet, shown as Case 8 in **Table 3**. Further, pre-training an RN50 model with the PlantCLEF2022 dataset in a self-supervised manner is also encouraged to distinguish the impact of convolution neural networks (CNNs) and vision transformers (ViTs), shown as Case 3 in **Table 3**. Simultaneously, fine-tuning a MoCo-v2 model in the PlantCLEF2022 dataset is also inspired to see the difference between CNN and ViT, shown as Case 5 in **Table 3**, even if we expect a lower performance because MoCo-v2 has a lower accuracy in ImageNet than MAE. However, training these models is too expensive. It is estimated that pre-training a ViT-large model as MAE costs more than *five* months with our current computation devices, four RTX 3090 GPUs. Therefore, these possible strategies are left for future studies.

**Implementation details.** As mentioned in Section 2.3, we have used the pre-trained ViT-L model from our previous paper (Xu et al., 2022c). Hence, we only focus on the last fine-tuning process in this paper, i.e. fine-tuning the ViT-L model in the plant disease recognition dataset. The ViT-L model has 24 transformer blocks with a hidden size of 1024, an MLP size of 4096, and 16 heads for each multi-head attention layer. The ViT-L model has approximately 307 million trainable parameters in total.

For a fair comparison, all models or transfer learning strategies were executed with the same settings with most of them following the fine-tuning schemes in MAE (He et al., 2022). In detail, the basic learning rate *lr_b_* was 0.001, and the actual learning *lr_a_* = *lr_b_* * *batch*/256 where *batch* was the batch size for different training dataset modes. The model was warmed up in 5 epochs with the learning rate increasing linearly from the first epoch to the set learning rate. Furthermore, 0.05 weight decay and 0.65 layer decay were utilized. Mixup (Zhang et al., 2017) and CutMix (Yun et al., 2019) were adopted as data augmentation methods.

The main change from MAE experimental setting was the batch size. Considering the number of images in each dataset, in the generic case, the batch size was 64 for CGIARWheat, Strawberry2021, CitrusLeaf, and TaiwanTomato, while it was 128 for other datasets. In terms of the few-shot case, the number of classes was one factor to set as the batch size should not be larger than the number of classes in the 1-shot case. Specifically, the batch size was 4 for most of the datasets, except for CGIARWheat with 2, IVADLTomato with 8, PlantDocCls with 16, PlantVillage with 32, and Rice1426 with 8. Besides, the generic case was trained with four GPUs while the few-shot cases were trained with only one GPU. To evaluate during thetraining process, the models were trained for 50 epochs and validated after every 5 epochs in the validation dataset, including the first epoch. The best models were tested in the testing datasets.

**Evaluation metric.** *Accuracy*, a common evaluation metric for image classification (Dosovitskiy et al., 2020; Xu et al., 2022b; He et al., 2022) was leveraged to assess different methods in a specific dataset. Since we aim to achieve versatile plant disease recognition performance, the *mean accuracy*, *mAcc*, over all datasets was utilized and computed as follows:


(1) $$mAcc=\frac{1}{M}\sum_{i=1}^{M}Acc_{i},$$

where *Acc_i_* is the testing accuracy in the *i*-th dataset and *N* is the total number of datasets. To assess the generality, testing accuracy and mean testing accuracy was employed, instead of validation accuracy and mean validation accuracy as used in MAE (He et al., 2022). In general, high testing accuracy and mean testing accuracy were desired.

### 3.2 Experimental results

#### 3.2.1 Main result

**Figure 7:**
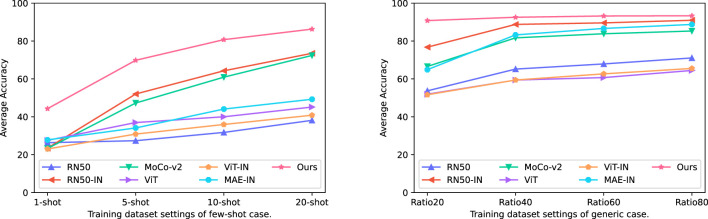
Curves of average testing accuracy *mAcc* of different methods in various training dataset modes over the 12 plant disease datasets.

As our main objective was achieving versatile plant disease recognition with a limited dataset, we first compared our method to other strategies. **Table 4** displays the mean testing accuracy of different methods over the 12 plant disease datasets mentioned in **Table 1** and **Figure 7** illustrates the tendency of mean testing accuracy of various methods in few-shot case and generic case respectively. The testing accuracy, the curve of validation loss, and the accuracy for each dataset can be found in the **Supplementary Material**. As shown in **Table 4**, the experimental results suggested that our method surpasses other methods by a clear margin across all dataset modes. Specifically, our method achieves 86.29 *mAcc* in a 20-shot case where only 20 images per class are utilized to train the models, compared to the second-best method, RN50-IN. We observed that the gap between our method and other methods becomes less when the number of training images increases. For example, the gap between our method and the second-best method, RN50-IN, in Ratio20 is 14.02 and becomes 2.37 in Ratio80, which suggests that a limited training dataset is one main obstacle for current methods.

**Table 4:** The mean testing accuracy *mAcc* of different training methods over the 12 datasets for plant disease recognition detailed in **Table 1**. .

	1-shot	5-shot	10-shot	20-shot	Ratio20	Ratio40	Ratio60	Ratio80
RN50 RN50-IN MoCo-v2 ViT ViT-IN MAE Ours	26.33 23.46 23.28 27.56 23.02 27.81 **44.28**	27.38 52.03 47.27 36.96 30.87 34.11 **69.83**	31.75 64.28 60.93 40.01 35.94 44.08 **80.73**	38.13 73.53 72.38 45.14 40.83 49.26 **86.29**	53.71 76.77 66.58 51.93 51.64 64.90 **90.79**	65.19 88.78 81.68 59.40 59.42 83.23 **92.55**	67.91 89.58 83.84 60.71 62.67 86.65 **93.23**	71.07 90.97 85.28 64.46 65.53 88.76 **93.34**

The best average accuracy for each dataset mode is in boldface.

In terms of the impact of transfer learning, the CNN-based method, RN50-IN, has the second-best mean testing accuracy, much higher than its counterpart, RN50 training from scratch, in the target dataset. However, ViT-IN shows its inferiority for a limited training dataset while more training images lead to a minor increase. We postulate that ViT is harder to train than the original ViT-IN, as suggested in the original paper (Dosovitskiy et al., 2020). In contrast, CNN has been regularly developed in the last decade, and thus the optimizing problem has been largely mitigated. A similar phenomenon exists in the loss function to train the models. For example, MoCo-v2 (Chen X. et al., 2020) scores 71.1 top-1 in accuracy in ImageNet while RN50 (He et al., 2016) obtains 77.15. On the contrary, MAE (He et al., 2022) achieves a 85.9 top-1 accuracy score. A comparison between ViT, ViT-IN, and MAE suggests that the self-supervised loss function contributes to the improvement of the ViT-based model in all training dataset modes.

Our method is based on MAE and is pre-trained one more time in the PlantCLEF2022 dataset. Excitingly, our method obtained 35.42, 36.65, and 37.03 higher accuracy scores than MAE in 5-shot, 10-shot, and 20-shot, respectively. The soar of the mean testing accuracy of our method compared to MAE proves that PlantCLEF2022 is essentially beneficial for achieving versatile plant disease recognition with a limited dataset. Our method not only achieved the best performance but also converged faster than other methods. For example, the validation loss was minimized to a low value within 5 epochs for the Ratio40 case. Please refer to **Figures S1** and **S2 in the Supplementary Material**.

Finally, 10 images for each class are available in PDD271* (Liu et al., 2021) and we used them as a few-shot learning task. Our method achieved a testing accuracy of 81.9 with only 1,355 images for both training and testing, compared to the original accuracy of 85.4 with 154,701 and 21,889 images for training and testing (Liu et al., 2021).

#### 3.2.2 Beyond plant disease

Beyond achieving versatile plant disease recognition, we believe that our transfer learning strategy is also beneficial for other types of plant-related work. We performed two types of experiments over two datasets. The Strawberry2021^7^ dataset, designed to predict plant growth stages, such as the young leaves and flowering stages, includes 557 images and 4 classes. The CottonWeedID15 (Chen et al., 2022) dataset requires the model to distinguish 15 types of weed in a cotton field, with 5,187 images in total.

The mean testing accuracy is displayed in **Table 5** while the details can be found in the **Supplementary Material**. It is interesting that our method scored a mean testing accuracy of 97.60 in a 5-shot case where only 5 images of each label were utilized to train the network. The current popular strategy obtains similar results but in the Ratio40 case, with approximately 121 images per class. The experimental results suggest that our method can also contribute to plant-related applications beyond plant disease recognition with few training samples.

**Table 5:** The mean testing accuracy of different training methods over Strawberry2021 and CottonWeedID15.

	1-shot	5-shot	10-shot	20-shot	Ratio20	Ratio40	Ratio60	Ratio80
RN50	20.50	21.75	26.45	35.95	39.90	68.90	66.90	78.25
RN50-IN	45.55	75.95	87.90	87.15	60.85	98.00	98.35	98.55
MoCo-v2	45.65	70.25	84.65	86.05	66.90	96.45	96.20	97.50
ViT	32.70	39.90	44.30	51.45	56.25	65.65	75.40	80.90
ViT-IN	27.20	33.35	43.10	45.25	55.05	68.30	75.50	82.35
MAE	17.45	41.45	59.50	59.20	85.20	97.80	98.35	98.75
Ours	**73.90**	**97.60**	**97.55**	**97.85**	**99.80**	**99.35**	**98.80**	**99.70**

The best average accuracy for each dataset mode shows in boldface.

#### 3.2.3 Discussion

**Limited data** is one main challenge in achieving high performance in the computer vision field (Xu et al., 2022a) and plant disease recognition (Lu et al., 2022; Xu et al., 2022b). Through our experimental results, we argue that the required amount of training dataset is partly dependent on the model or pre-trained model. As shown in **Table 4**, the mean testing accuracy of RN50-IN was 83.23 in the Ratio40 case and gains 12.76 from the Ratio20 case, while our method only had a 1.76 increase. Through this analysis, we believe that our method mitigates the requirement of a large dataset for plant disease recognition.

Furthermore, we emphasized that more training data tends to contribute to high performance but the gains become lower when a decent performance is obtained. For example, 20 percent more data only resulted in an increase of 0.11 in mean testing accuracy score in the Ratio60 case with our strategy. Therefore, recognizing the limitation of increasing data is also essential for practical applications. Sometimes, we may have to resort to alternative ways to have higher performance, instead of just increasing the training dataset.

**Future work.** First, we emphasize here that we are not aiming to achieve the best performance with our method in this paper. Instead, we propose a versatile plant disease recognition method with a limited training dataset. Therefore, we encourage our method to be used as a baseline for future works, although we did obtain superior performance in plant disease recognition. For example, is the PlantCLEF2022 dataset beneficial for a CNN-based network? In this way, we can pre-train the RN50 model and then fine-tune it in the target dataset. Moreover, it is interesting to analyze the reason why the same model and strategy behave differently in different datasets. For example, our method achieved a score of 97.4 in testing accuracy in the 20-shot case in the PlantVillage dataset as shown in **Table S1** while scoring only 63.8 in the IVADLTomato dataset as shown in **Table S9**. Furthermore, we only validated our method in plant disease recognition, and encourage deploying our method to perform object detection and segmentation (Xu et al., 2022b). We also highlight combining our transfer learning with other unsupervised or self-supervised learning in the future. For instance, using a few labeled images to train a model and then leveraging the trained model to generate pseudo labels for unlabeled images (Li and Chao, 2021) and reduce annotation cost. Our preliminary results in Strawberry2021 and CottonWeedID15 suggest that our transfer learning strategy is not just promising for plant disease but also plant stage recognition and weed identification. We encourage more plant-related applications to deploy our method as a baseline.

## 4 Conclusion

We proposed a simple but nontrivial transfer learning strategy to achieve versatile plant disease recognition with limited data. Our method strikingly outperforms current strategies, not only on 12 plant disease recognition datasets but also in one plant growth stage prediction and one weed detection dataset. One main characteristic of our method is the use of PlantCLEF2022, a plant-related dataset including 2,885,052 images and 80,000 classes with huge image variations, which enables our transfer learning to be beneficial for versatile plant disease recognition tasks. Considering the large-scale dataset, our method employs a vision transformer (ViT) model because of its higher performance than the widely used convolution neural network. To reduce the computation cost, dual transfer learning is leveraged as the ViT model is first pre-trained with ImageNet in a self-supervised manner because the ImageNet dataset is different to the plant disease dataset. The model is then fine-tuned with PlantCLEF2022 in a supervised manner. We believe that our transfer learning strategy contributes to the field and to fuel the community, our codes and the pre-trained model are publicly available.

## Data availability statement

Publicly available datasets were analyzed in this study. Their download links can be found here: https://github.com/xml94/MAE_plant_disease.

## Author contributions

MX: conceptualization, methodology, software, writing - original draft, writing - review and editing. SY: supervision and writing - review and editing. YJ: writing - review and editing. DP: supervision, project administration, funding acquisition, writing - review and editing. All authors contributed to the article and approved the submitted version.

## Funding

This research is partly supported by Basic Science Research Program through the National Research Foundation of Korea (NRF) funded by the Ministry of Education (No.2019R1A6A1A09031717), supported by the National Research Foundation of Korea(NRF) grant funded by the Korean government (MSIT). (NRF-2021R1A2C1012174), and supported by the Korea Institute of Planning and Evaluation for Technology in Food, Agriculture, and Forestry (IPET) and the Korea Smart Farm R&D Foundation (KosFarm) through the Smart Farm Innovation Technology Development Program, funded by the Ministry of Agriculture, Food and Rural Affairs (MAFRA) and the Ministry of Science and ICT (MSIT), Rural Development Administration (RDA) (No. 421005-04).

## Acknowledgments

We appreciated the valuable suggestions from the reviewers to make the paper clear and easier to follow.

## Conflict of interest

The authors declare that the research was conducted in the absence of any commercial or financial relationships that could be construed as a potential conflict of interest.

## Publisher’s note

All claims expressed in this article are solely those of the authors and do not necessarily represent those of their affiliated organizations, or those of the publisher, the editors and the reviewers. Any product that may be evaluated in this article, or claim that may be made by its manufacturer, is not guaranteed or endorsed by the publisher.

## Footnotes

2
https://data.mendeley.com/datasets/ngdgg79rzb/1

3
https://github.com/IVADL/tomato-disease-detector

4
https://www.kaggle.com/datasets/shadabhussain/cgiar-computer-vision-for-crop-disease?resource=download

5
https://github.com/xml94/MAE_plant_disease/blob/main/visualize_dataset/dataset.md

6
https://www.aicrowd.com/challenges/lifeclef-2022-plant

7
https://aistudio.baidu.com/aistudio/datasetdetail/98233

## References

[B1] Abade A. Ferreira P. A. de Barros Vidal F. (2021). Plant diseases recognition on images using convolutional neural networks: A systematic review. Comput. Electron. Agric. 185, 106125. doi: 10.1016/j.compag.2021.106125

[B2] Abbas A. Jain S. Gour M. Vankudothu S. (2021). Tomato plant disease detection using transfer learning with c-gan synthetic images. Comput. Electron. Agric. 187, 106279. doi: 10.1016/j.compag.2021.106279

[B3] Afifi A. Alhumam A. Abdelwahab A. (2020). Convolutional neural network for automatic identification of plant diseases with limited data. Plants 10, 28. doi: 10.3390/plants10010028 33374398 PMC7823428

[B4] Azizi S. Mustafa B. Ryan F. Beaver Z. Freyberg J. Deaton J. . (2021). “Big self-supervised models advance medical image classification,” in Proceedings of the IEEE/CVF international conference on computer vision, Montreal: IEEE. 3478–3488.

[B5] Barbedo J. G. A. (2019). Plant disease identification from individual lesions and spots using deep learning. Biosyst. Eng. 180, 96–107. doi: 10.1016/j.biosystemseng.2019.02.002

[B6] Chen J. Chen J. Zhang D. Sun Y. Nanehkaran Y. A. (2020). Using deep transfer learning for image-based plant disease identification. Comput. Electron. Agric. 173, 105393. doi: 10.1016/j.compag.2020.105393

[B7] Chen X. Fan H. Girshick R. He K. (2020). Improved baselines with momentum contrastive learning. arXiv. preprint. arXiv:2003.04297.

[B8] Chen D. Lu Y. Li Z. Young S. (2022). Performance evaluation of deep transfer learning on multi-class identification of common weed species in cotton production systems. Comput. Electron. Agric. 198, 107091. doi: 10.1016/j.compag.2022.107091

[B9] Deng J. Dong W. Socher R. Li L.-J. Li K. Fei-Fei L. (2009). “Imagenet: A large-scale hierarchical image database,” in 2009 IEEE conference on computer vision and pattern recognition (Miami Beach: Ieee), 248–255.

[B10] Dosovitskiy A. Beyer L. Kolesnikov A. Weissenborn D. Zhai X. Unterthiner T. . (2020). “An image is worth 16x16 words: Transformers for image recognition at scale,” in International conference on learning representations.

[B11] Egusquiza I. Picon A. Irusta U. Bereciartua-Perez A. Eggers T. Klukas C. . (2022). Analysis of few-shot techniques for fungal plant disease classification and evaluation of clustering capabilities over real datasets. Front. Plant Sci. 295. doi: 10.3389/fpls.2022.813237 PMC8959904 35356111

[B12] Fan X. Luo P. Mu Y. Zhou R. Tjahjadi T. Ren Y. (2022). Leaf image based plant disease identification using transfer learning and feature fusion. Comput. Electron. Agric. 196, 106892. doi: 10.1016/j.compag.2022.106892

[B13] He K. Chen X. Xie S. Li Y. Dollár P. Girshick R. (2022). “Masked autoencoders are scalable vision learners,” in Proceedings of the IEEE/CVF conference on computer vision and pattern recognition, New Orleans: IEEE. 16000–16009.

[B14] He K. Zhang X. Ren S. Sun J. (2016). “Deep residual learning for image recognition,” in Proceedings of the IEEE conference on computer vision and pattern recognition, Caesars Palace: IEEE. 770–778.

[B15] Hughes D. Salathé M . (2015). An open access repository of images on plant health to enable the development of mobile disease diagnostics. arXiv. preprint. arXiv:1511.08060.

[B16] Kim B. Han Y.-K. Park J.-H. Lee J. (2021). Improved vision-based detection of strawberry diseases using a deep neural network. Front. Plant Sci. 11, 559172. doi: 10.3389/fpls.2020.559172 33584739 PMC7874225

[B17] Kolesnikov A. Beyer L. Zhai X. Puigcerver J. Yung J. Gelly S. . (2020). “Big transfer (bit): General visual representation learning,” in European Conference on computer vision (Springer), 491–507.

[B18] Kornblith S. Shlens J. Le Q. V. (2019). “Do better imagenet models transfer better?,” in Proceedings of the IEEE/CVF conference on computer vision and pattern recognition, Long Beach: IEEE. 2661–2671.

[B19] Krizhevsky A. Sutskever I. Hinton G. E. (2012). “Imagenet classification with deep convolutional neural networks,” in Advances in neural information processing systems, Lake Tahoe vol. 25. Eds. Pereira F. Burges C. Bottou L. Weinberger K. (Curran Associates, Inc).

[B20] Li Y. Chao X. (2021). Semi-supervised few-shot learning approach for plant diseases recognition. Plant Methods 17, 1–10. doi: 10.1186/s13007-021-00770-1 34176505 PMC8237441

[B21] Liu X. Min W. Mei S. Wang L. Jiang S. (2021). Plant disease recognition: A large-scale benchmark dataset and a visual region and loss reweighting approach. IEEE Trans. Image. Process. 30, 2003–2015. doi: 10.1109/TIP.2021.3049334 33444137

[B22] Lu Y. Chen D. Olaniyi E. Huang Y. (2022). Generative adversarial networks (gans) for image augmentation in agriculture: A systematic review. Comput. Electron. Agric. 200, 107208. doi: 10.1016/j.compag.2022.107208

[B23] Mohanty S. P. Hughes D. P. Salathé M. (2016). Using deep learning for image-based plant disease detection. Front. Plant Sci. 7, 1419. doi: 10.3389/fpls.2016.01419 27713752 PMC5032846

[B24] Ngugi L. C. Abelwahab M. Abo-Zahhad M. (2021). Recent advances in image processing techniques for automated leaf pest and disease recognition–a review. Inf. Process. Agric. 8, 27–51. doi: 10.1016/j.inpa.2020.04.004

[B25] Olaniyi E. Chen D. Lu Y. Huang Y. (2022). Generative adversarial networks for image augmentation in agriculture: a systematic review. arXiv. preprint. arXiv:2204.04707.

[B26] Pan S. J. Yang Q. (2009). A survey on transfer learning. IEEE Trans. Knowledge. Data Eng. 22, 1345–1359. doi: 10.1109/TKDE.2009.191

[B27] Qian X. Zhang C. Chen L. Li K. (2022). Deep learning-based identification of maize leaf diseases is improved by an attention mechanism: Self-attention. Front. Plant Sci. 1154. doi: 10.3389/fpls.2022.864486 PMC9096888 35574079

[B28] Rahman C. R. Arko P. S. Ali M. E. Khan M. A. I. Apon S. H. Nowrin F. . (2020). Identification and recognition of rice diseases and pests using convolutional neural networks. Biosyst. Eng. 194, 112–120. doi: 10.1016/j.biosystemseng.2020.03.020

[B29] Ramcharan A. Baranowski K. McCloskey P. Ahmed B. Legg J. Hughes D. P. (2017). Deep learning for image-based cassava disease detection. Front. Plant Sci. 8, 1852. doi: 10.3389/fpls.2017.01852 29163582 PMC5663696

[B30] Rauf H. T. Saleem B. A. Lali M. I. U. Khan M. A. Sharif M. Bukhari S. A. C. (2019). A citrus fruits and leaves dataset for detection and classification of citrus diseases through machine learning. Data Brief 26, 104340. doi: 10.1016/j.dib.2019.104340 31516936 PMC6731382

[B31] Sethy P. K. Barpanda N. K. Rath A. K. Behera S. K. (2020). Deep feature based rice leaf disease identification using support vector machine. Comput. Electron. Agric. 175, 105527. doi: 10.1016/j.compag.2020.105527

[B32] Singh D. Jain N. Jain P. Kayal P. Kumawat S. Batra N. (2020). “Plantdoc: a dataset for visual plant disease detection,” in Proceedings of the 7th ACM IKDD CoDS and 25th COMAD, Hyderabad: ACM (Association for Computing Machinery). 249–253.

[B33] Thapa R. Wang Q. Snavely N. Belongie S. Khan A. (2021). The plant pathology 2021 challenge dataset to classify foliar disease of apples. doi: 10.1002/aps3.11390 PMC7526434 33014634

[B34] Thapa R. Zhang K. Snavely N. Belongie S. Khan A. (2020). The plant pathology challenge 2020 data set to classify foliar disease of apples. Appl. Plant Sci. 8, e11390. doi: 10.1002/aps3.11390 33014634 PMC7526434

[B35] Too E. C. Yujian L. Njuki S. Yingchun L. (2019). A comparative study of fine-tuning deep learning models for plant disease identification. Comput. Electron. Agric. 161, 272–279. doi: 10.1016/j.compag.2018.03.032

[B36] Tripuraneni N. Jordan M. Jin C. (2020). On the theory of transfer learning: The importance of task diversity. Adv. Neural Inf. Process. Syst. 33, 7852–7862. doi: 10.5555/3495724.3496382

[B37] Wang F. Rao Y. Luo Q. Jin X. Jiang Z. Zhang W. . (2022). Practical cucumber leaf disease recognition using improved swin transformer and small sample size. Comput. Electron. Agric. 199, 107163. doi: 10.1016/j.compag.2022.107163

[B38] Wu Z. Xiong Y. Yu S. X. Lin D. (2018). “Unsupervised feature learning via non-parametric instance discrimination,” in Proceedings of the IEEE conference on computer vision and pattern recognition, Salt Lake City: ACM (Association for Computing Machinery). 3733–3742.

[B39] Xing S. Lee H. J. (2022). Crop pests and diseases recognition using danet with tldp. Comput. Electron. Agric. 199, 107144. doi: 10.1016/j.compag.2022.107144

[B40] Xu M. Yoon S. Fuentes A. Park D. S. (2022a). A comprehensive survey of image augmentation techniques for deep learning. arXiv. preprint. arXiv:2205.01491. Bologna

[B41] Xu M. Yoon S. Fuentes A. Yang J. Park D. S. (2022b). Style-consistent image translation: A novel data augmentation paradigm to improve plant disease recognition. Front. Plant Sci. 12, 773142–773142. doi: 10.3389/fpls.2021.773142 35197989 PMC8858820

[B42] Xu M. Yoon S. Jeong Y. Lee J. Park D. S. (2022c). “Transfer learning with self-supervised vision transformer for large-scale plant identification,” in International conference of the cross-language evaluation forum for European languages (Springer), 2253–2261.

[B43] Yadav A. Thakur U. Saxena R. Pal V. Bhateja V. Lin J. C.-W. (2022). Afd-net: Apple foliar disease multi classification using deep learning on plant pathology dataset. Plant Soil, 477, 1–17. doi: 10.1007/s11104-022-05407-3

[B44] Yang G. He Y. Yang Y. Xu B. (2020). Fine-grained image classification for crop disease based on attention mechanism. Front. Plant Sci. 11, 600854. doi: 10.3389/fpls.2020.600854 33414798 PMC7783357

[B45] Yun S. Han D. Oh S. J. Chun S. Choe J. Yoo Y. (2019). “Cutmix: Regularization strategy to train strong classifiers with localizable features,” in Proceedings of the IEEE/CVF international conference on computer vision, Long Beach: IEEE. 6023–6032.

[B46] Zhang H. Cisse M. Dauphin Y. N. Lopez-Paz D. (2017). Mixup: Beyond empirical risk minimization. arXiv. preprint. arXiv:1710.09412.

[B47] Zhao X. Li K. Li Y. Ma J. Zhang L. (2022). Identification method of vegetable diseases based on transfer learning and attention mechanism. Comput. Electron. Agric. 193, 106703. doi: 10.1016/j.compag.2022.106703

