# Supplementary material for: Transfer learning for versatile plant disease recognition with limited data
Source: Front Plant Sci. 2022 Nov 23;13:1010981. doi: 10.3389/fpls.2022.1010981 (PMC9726777; doi:10.3389/fpls.2022.1010981)
Supplement: Supplementary file 1 [file DataSheet_1.pdf]

Transfer Learning for Versatile Plant Disease Recognition with Limited Data Supplementary Data

1 MORE EXPERIMENTAL RESULTS

We give all the curves of validation accuracy and loss function related to plant disease recognition from Figure S1 to S12. Simultaneously, the testing accuracy in different datasets with different method is displayed from Table S1 to S12.

Beyond plant disease, the curves in Strawberry2021 and CottonWeedID15 are shown from Figure S13 to S14. The Strawberry2021 dataset is related to strawberry growth stage while the CottonWeedID15 is designed to recongize the weeds in the cotton filed. The testing accuracy for the two datasets is given in Table S13 and S14.

	1-shot	5-shot	10-shot	20-shot	Ratio20	Ratio40	Ratio60	Ratio80
RN50	3.6	14.9	34.2	46.1	95.0	98.2	99.2	99.4
RN50-IN	21.6	76.7	86.4	93.3	99.3	99.7	99.7	99.7
MoCo-v2	19.7	54.5	77.3	90.0	98.9	99.6	99.7	99.7
ViT	11.3	19.0	37.2	46.8	93.7	97.1	98.5	98.7
ViT-IN	5.9	18.3	32.9	43.5	94.0	97.2	98.5	98.7
MAE	4.8	26.1	69.8	88.1	99.5	99.8	99.8	99.9
Ours	46.9	89.4	93.9	97.4	99.7	99.9	99.9	99.9

Table S1. The testing accuracy of different training methods on PlantVillage.

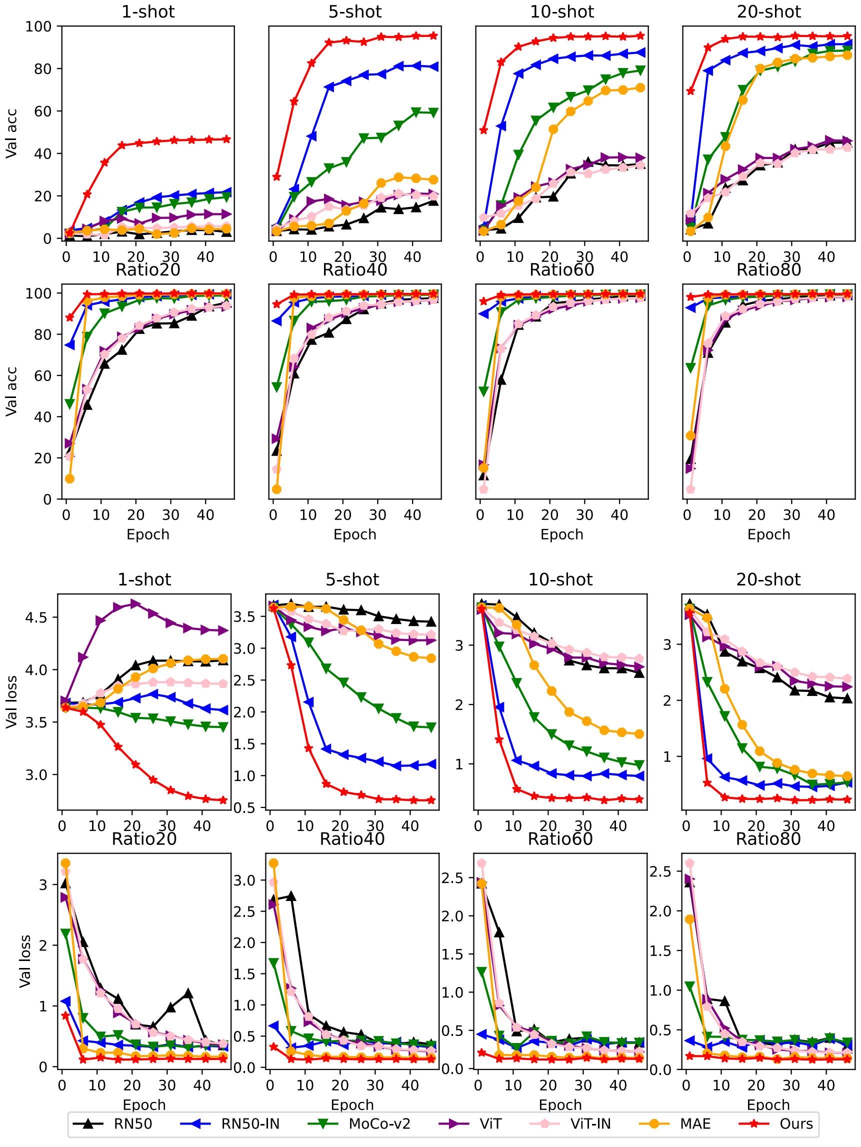

Figure S1. The curves of validation accuracy and loss on PlantVillage.

	1-shot	5-shot	10-shot	20-shot	Ratio20	Ratio40	Ratio60	Ratio80
RN50	4.7	4.7	6.8	8.1	5.9	13.6	11.4	16.1
RN50-IN	14.4	33.9	44.5	53.8	50.4	62.7	69.9	68.6
MoCo-v2	12.7	27.1	38.1	53.4	22.5	41.5	52.6	54.7
ViT	5.1	10.2	8.5	8.1	6.8	10.2	8.1	11.9
ViT-IN	5.1	8.5	7.2	10.6	6.8	7.6	11.0	12.3
MAE	3.8	14.8	21.2	42.8	37.3	65.3	69.9	72.5
Ours	30.5	57.2	70.8	75.0	72.0	73.3	78.4	78.0

Table S2. The testing accuracy of different training methods on PlantDocCls.

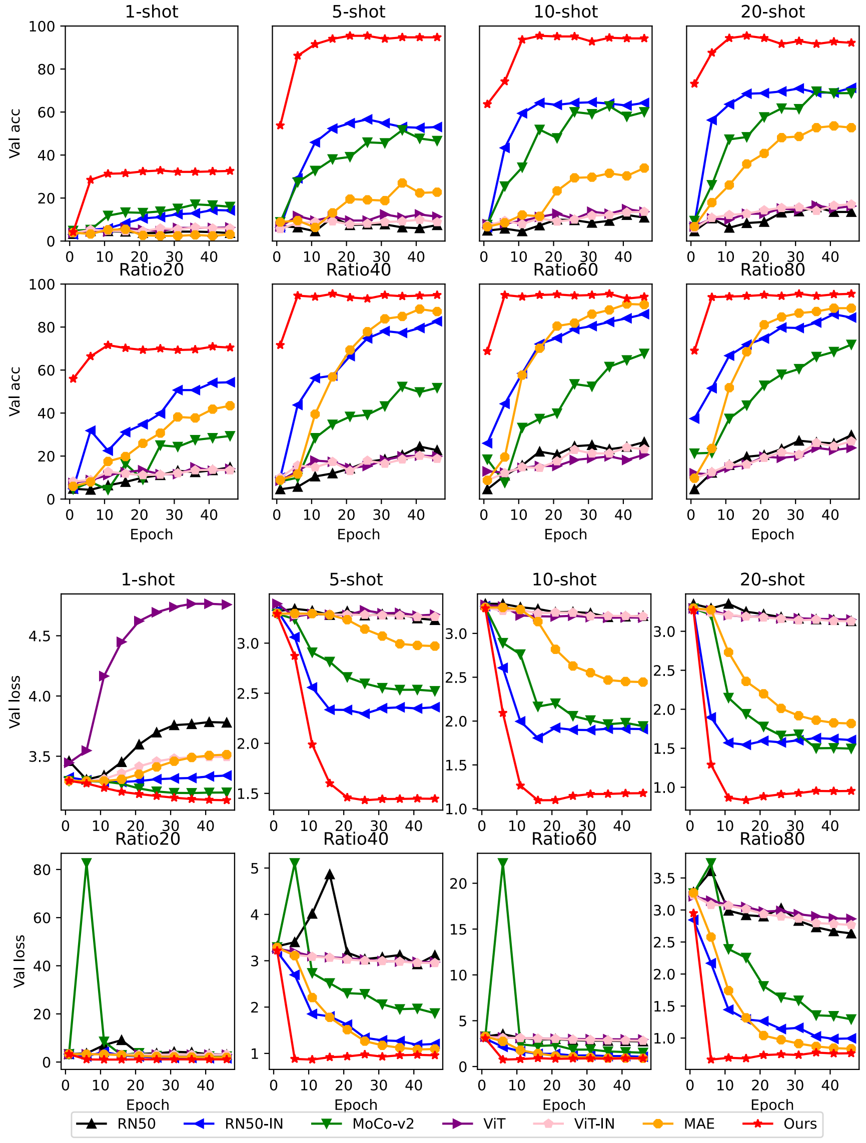

Figure S2. The curves of validation accuracy and loss on PlantDocCls.

	1-shot	5-shot	10-shot	20-shot	Ratio20	Ratio40	Ratio60	Ratio80
RN50	61.3	43.6	33.4	36.8	69.8	75.4	77.8	79.9
RN50-IN	23.1	37.8	53.6	51.1	86.7	87.1	87.5	88.2
MoCo-v2	22.1	39.4	51.5	54.5	82.4	84.6	86.0	86.3
ViT	44.8	40.6	36.5	32.2	66.1	69.6	71.5	72.7
ViT-IN	31.9	19.8	46.2	42.3	65.5	69.1	71.3	72.6
MAE	61.5	34.1	62.0	27.5	88.8	88.3	88.7	89.0
Ours	60.8	66.9	67.1	78.9	88.1	88.6	88.9	88.9

Table S3. The testing accuracy of different training methods on Cassava.

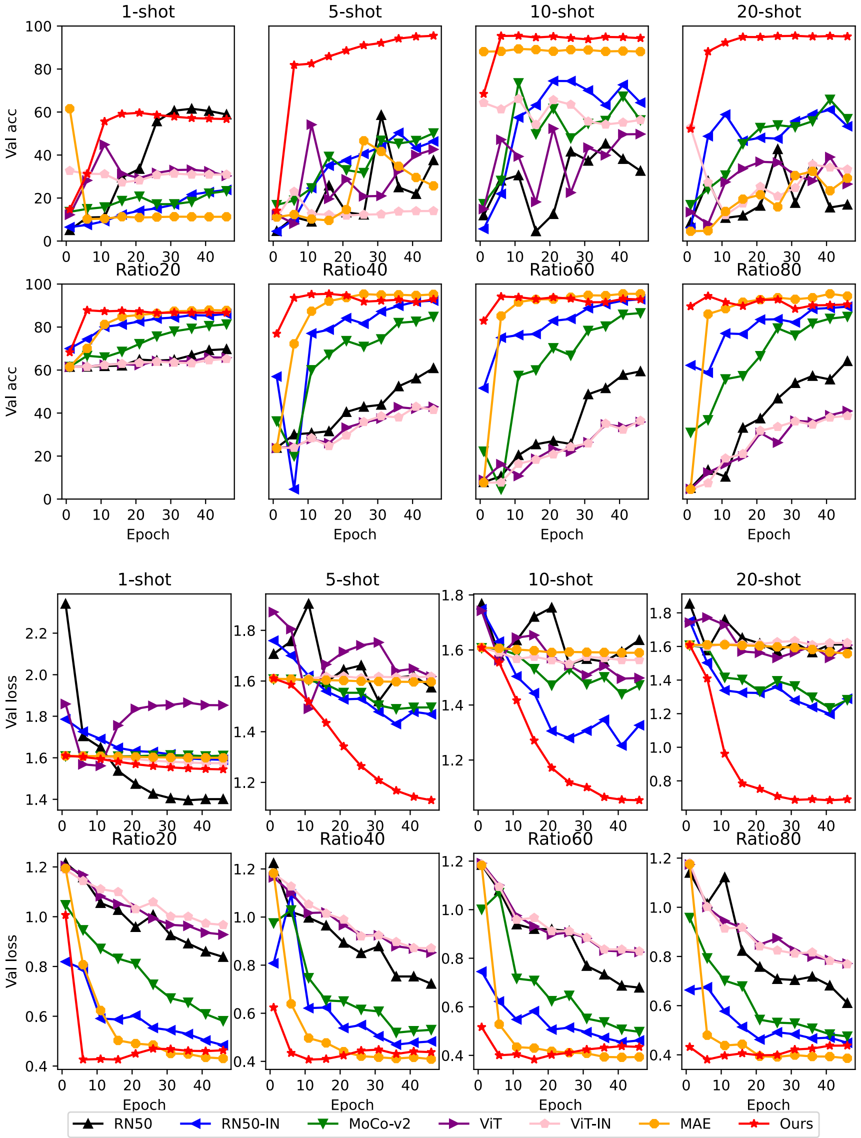

Figure S3. The curves of validation accuracy and loss on Cassava.

	1-shot	5-shot	10-shot	20-shot	Ratio20	Ratio40	Ratio60	Ratio80
RN50	35.3	32.5	36.4	37.2	44.9	44.0	46.8	59.1
RN50-IN	27.8	57.9	70.5	87.1	89.5	93.1	95.9	91.7
MoCo-v2	26.2	51.2	65.0	82.1	83.1	91.4	92.3	93.9
ViT	35.0	34.4	36.4	36.1	43.2	41.0	41.6	46.4
ViT-IN	36.9	34.4	35.0	37.7	36.3	39.3	41.3	51.4
MAE	35.5	32.5	35.3	37.7	34.3	91.4	94.2	95.0
Ours	55.9	67.2	82.6	91.2	96.1	94.7	97.5	96.1

Table S4. The testing accuracy of different training methods on Apple2020.

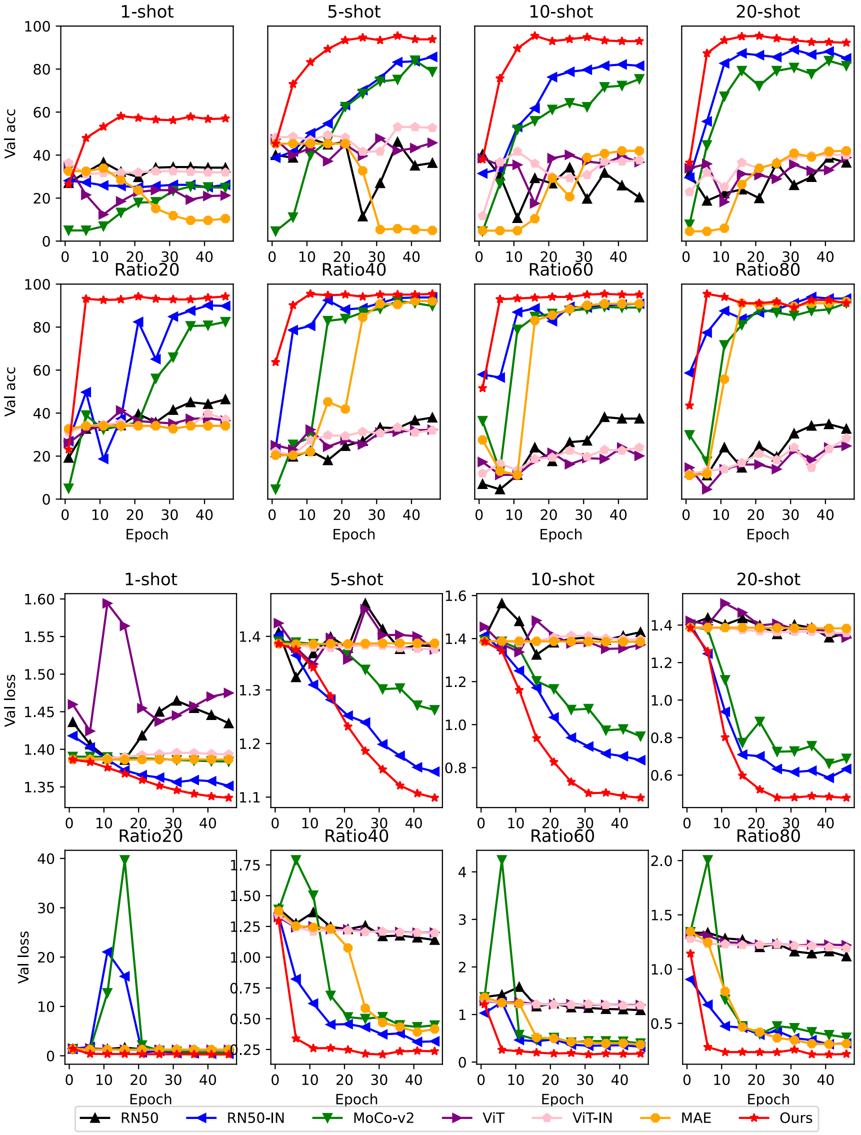

Figure S4. The curves of validation accuracy and loss on Apple2020.

	1-shot	5-shot	10-shot	20-shot	Ratio20	Ratio40	Ratio60	Ratio80
RN50	25.9	25.9	25.9	25.9	52.8	77.0	88.7	88.8
RN50-IN	23.2	39.6	48.1	68.7	92.7	92.3	94.7	94.1
MoCo-v2	19.0	44.9	51.0	66.8	91.2	91.5	93.9	93.2
ViT	26.7	25.6	28.8	37.8	41.1	58.6	66.7	73.0
ViT-IN	24.3	28.1	24.7	33.3	46.9	55.8	63.4	72.5
MAE	25.9	24.8	28.5	32.5	93.1	93.6	95.0	95.0
Ours	38.4	64.6	76.2	88.2	95.9	95.7	96.3	96.6

Table S5. The testing accuracy of different training methods on Apple2021.

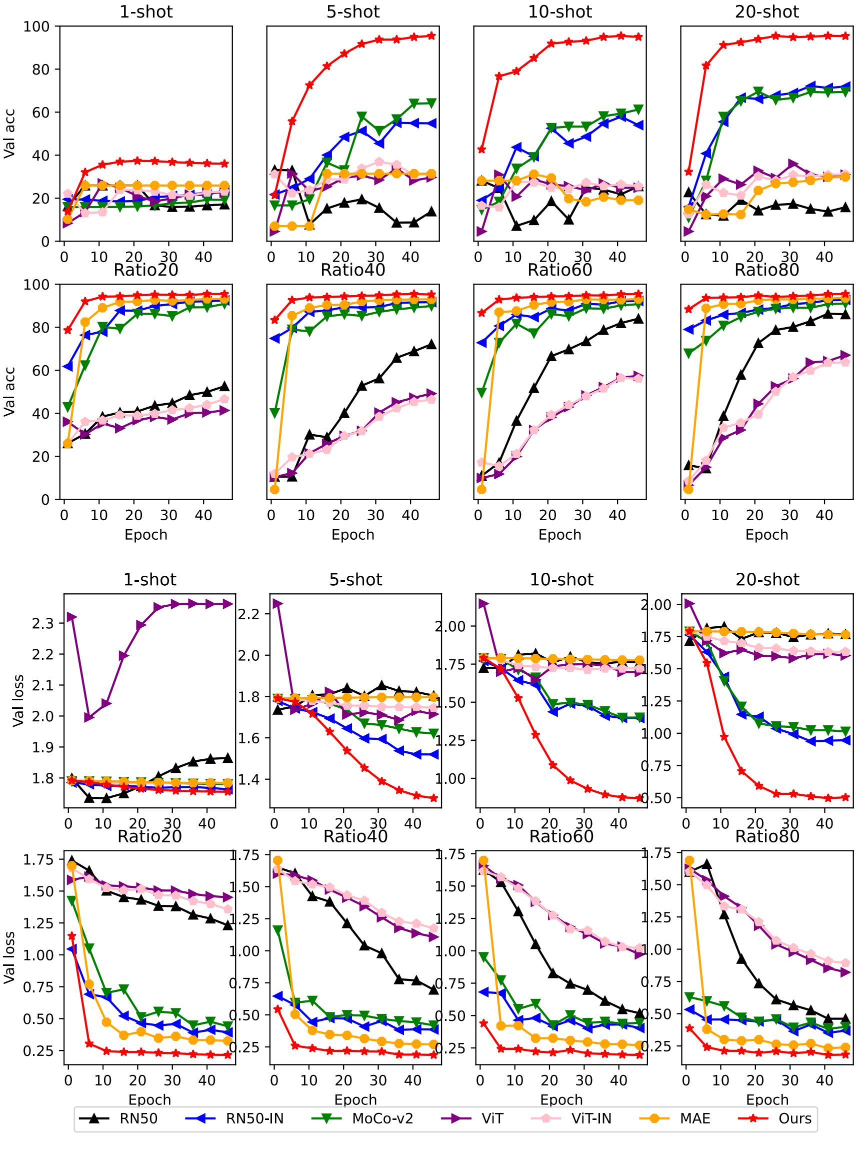

Figure S5. The curves of validation accuracy and loss on Apple2021.

	1-shot	5-shot	10-shot	20-shot	Ratio20	Ratio40	Ratio60	Ratio80
RN50	43.1	23.0	41.4	42.5	44.0	61.4	64.1	71.9
RN50-IN	16.1	27.6	48.9	63.8	89.2	94.2	96.4	97.8
MoCo-v2	19.5	24.7	49.4	59.2	48.4	70.4	68.7	85.6
ViT	38.5	53.4	52.9	51.1	41.2	40.4	45.6	50.4
ViT-IN	20.7	43.7	47.7	45.4	38.6	40.1	46.6	52.5
MAE	40.8	46.6	52.3	58.0	64.3	93.5	94.7	94.2
Ours	35.1	62.1	81.6	83.9	97.1	99.6	100.0	99.3

Table S6. The testing accuracy of different training methods on Rice1426.

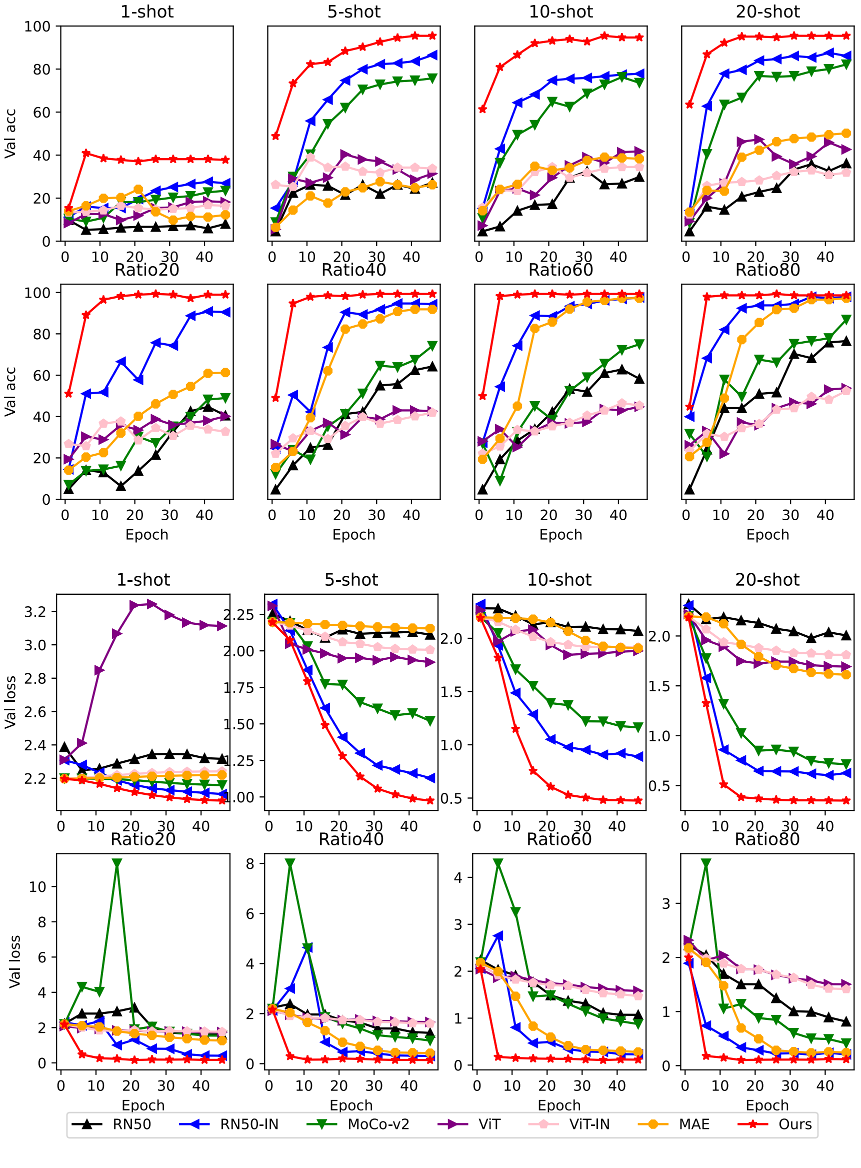

Figure S6. The curves of validation accuracy and loss on Rice1426.

	1-shot	5-shot	10-shot	20-shot	Ratio20	Ratio40	Ratio60	Ratio80
RN50	31.2	42.5	45.7	46.5	88.3	94.2	97.3	99.0
RN50-IN	33.9	69.2	69.8	77.0	98.7	100.0	99.9	99.8
MoCo-v2	43.5	66.6	64.2	79.3	97.3	99.5	100.0	100.0
ViT	43.0	60.4	55.1	69.0	86.6	94.9	97.3	98.6
ViT-IN	40.0	45.0	49.3	62.7	88.8	96.2	98.1	99.2
MAE	44.5	61.3	64.3	59.4	100.0	100.0	100.0	100.0
Ours	67.9	85.4	90.1	92.4	100.0	100.0	100.0	100.0

Table S7. The testing accuracy of different training methods on Rice5932.

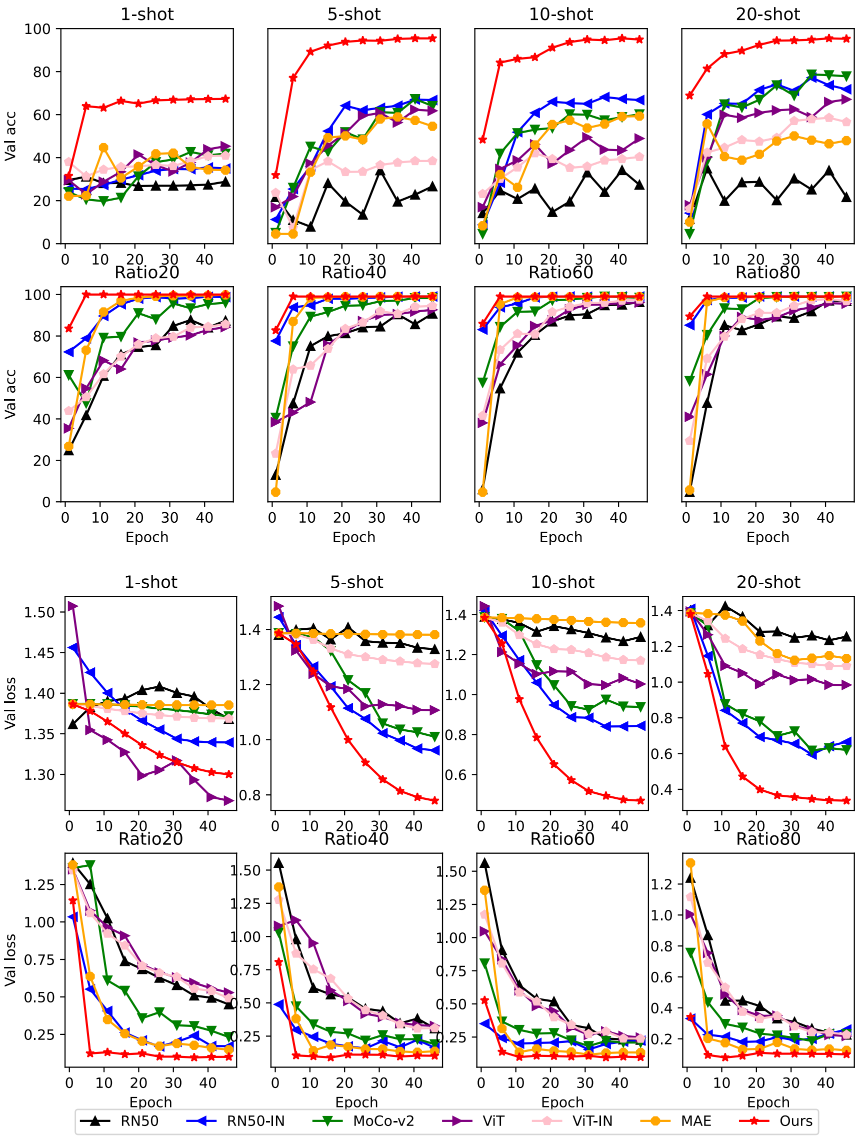

Figure S7. The curves of validation accuracy and loss on Rice5932.

	1-shot	5-shot	10-shot	20-shot	Ratio20	Ratio40	Ratio60	Ratio80
RN50	9.6	20.3	22.8	33.8	25.2	44.1	48.8	52.0
RN50-IN	23.5	70.5	7.2	82.9	15.7	66.9	59.8	78.0
MoCo-v2	22.8	56.6	65.8	79.0	17.3	52.8	55.1	59.1
ViT	20.6	28.8	38.1	44.1	23.6	42.5	46.5	51.2
ViT-IN	13.9	29.9	30.6	28.1	29.1	47.2	49.6	52.8
MAE	20.6	21.4	36.7	51.6	25.2	33.1	52.8	59.1
Ours	41.3	75.4	86.8	93.6	62.2	74.0	78.7	85.0

Table S8. The testing accuracy of different training methods on TaiwanTomato.

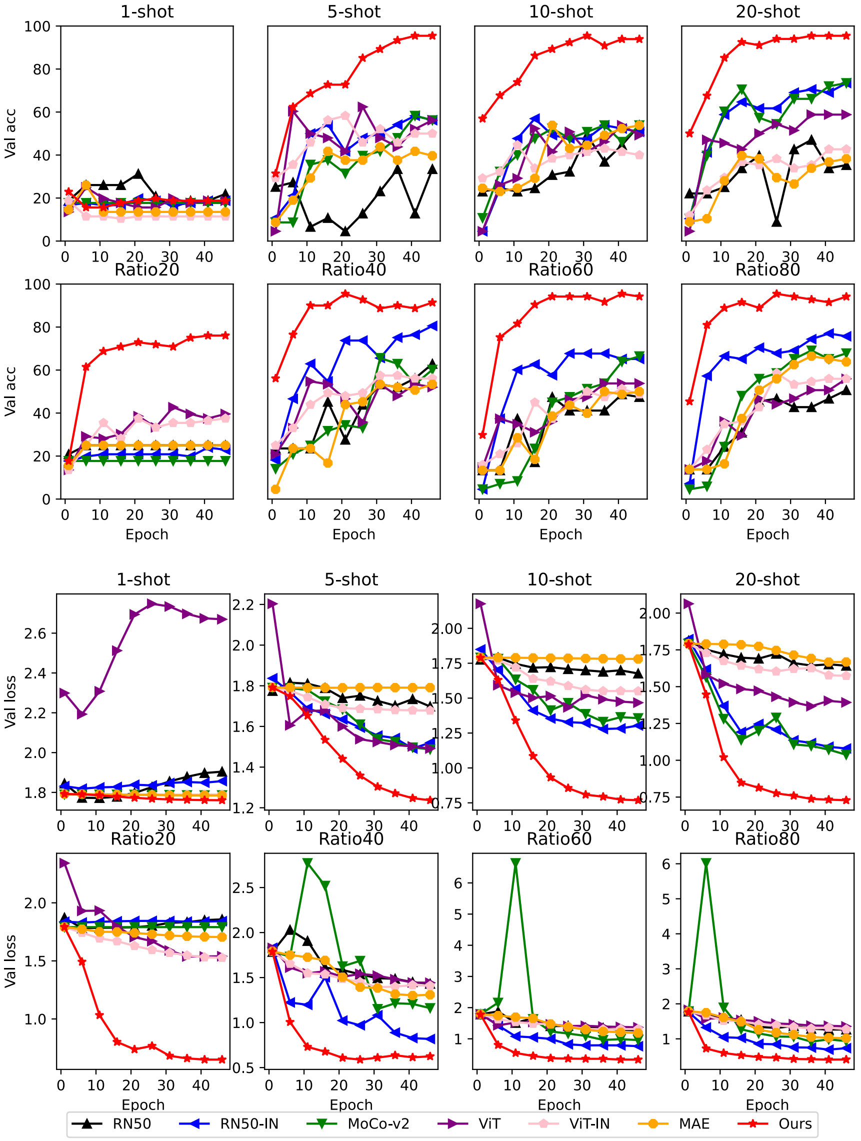

Figure S8. The curves of validation accuracy and loss on TaiwanTomato.

	1-shot	5-shot	10-shot	20-shot	Ratio20	Ratio40	Ratio60	Ratio80
RN50	22.8	26.8	30.7	35.4	63.5	64.5	68.8	70.0
RN50-IN	17.3	33.1	38.6	52.8	91.6	96.8	97.3	96.6
MoCo-v2	17.3	34.6	37.0	52.0	76.6	86.1	91.7	94.6
ViT	16.5	28.3	35.4	40.9	60.0	56.9	60.8	64.0
ViT-IN	11.0	26.8	27.6	33.9	60.9	57.5	62.9	65.0
MAE	25.2	29.1	29.9	32.3	53.7	95.0	97.5	97.3
Ours	19.7	40.9	52.8	63.8	97.5	98.7	98.8	98.7

Table S9. The testing accuracy of different training methods on IVADLTomato.

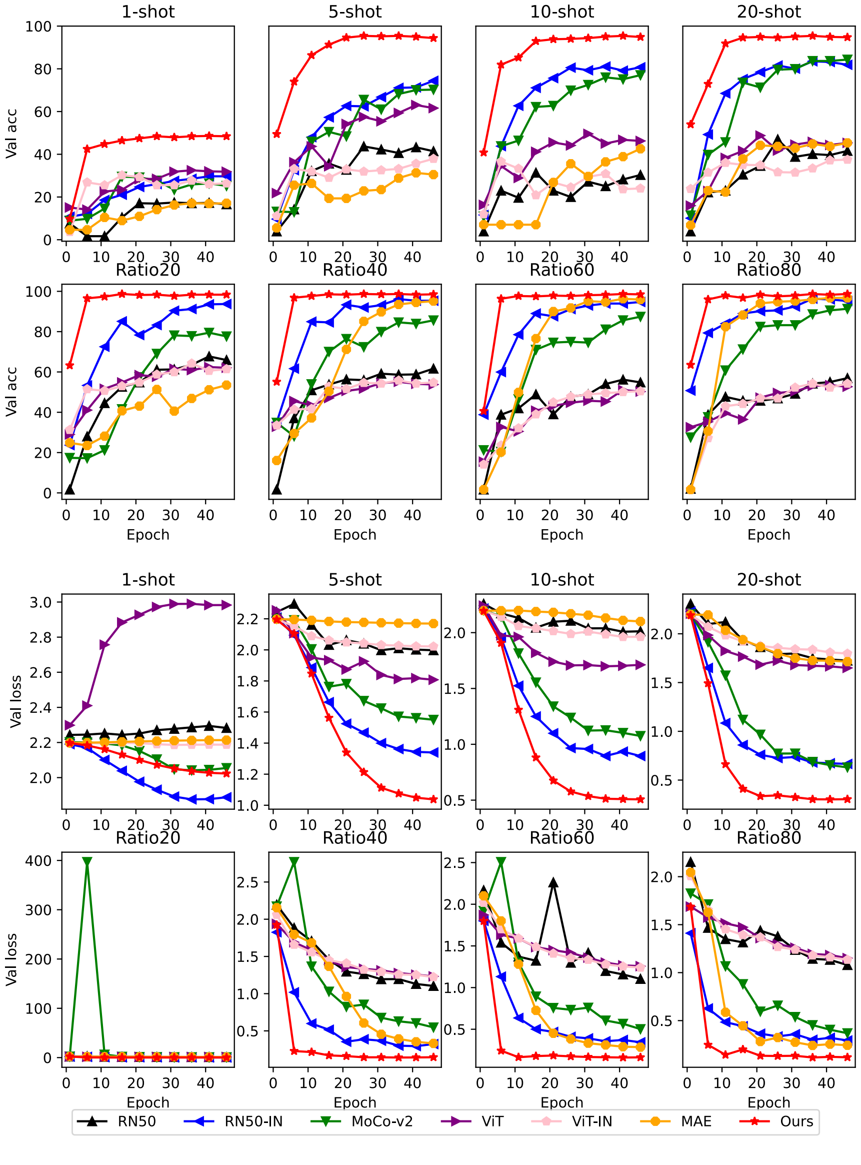

Figure S9. The curves of validation accuracy and loss on IVADLTomato.

	1-shot	5-shot	10-shot	20-shot	Ratio20	Ratio40	Ratio60	Ratio80
RN50	17.7	32.0	27.8	48.3	65.1	75.6	74.4	75.0
RN50-IN	30.0	53.0	79.0	82.7	92.8	95.7	96.8	97.4
MoCo-v2	27.3	54.5	73.3	83.7	84.0	93.1	93.4	93.3
ViT	28.3	47.5	44.2	52.0	58.8	66.2	62.0	65.1
ViT-IN	26.0	27.8	35.2	40.7	57.9	67.1	63.5	66.0
MAE	17.2	21.5	39.8	44.0	88.3	94.9	96.0	95.5
Ours	48.8	68.0	90.2	95.5	98.7	97.4	97.1	98.4

Table S10. The testing accuracy of different training methods on IVADLRose.

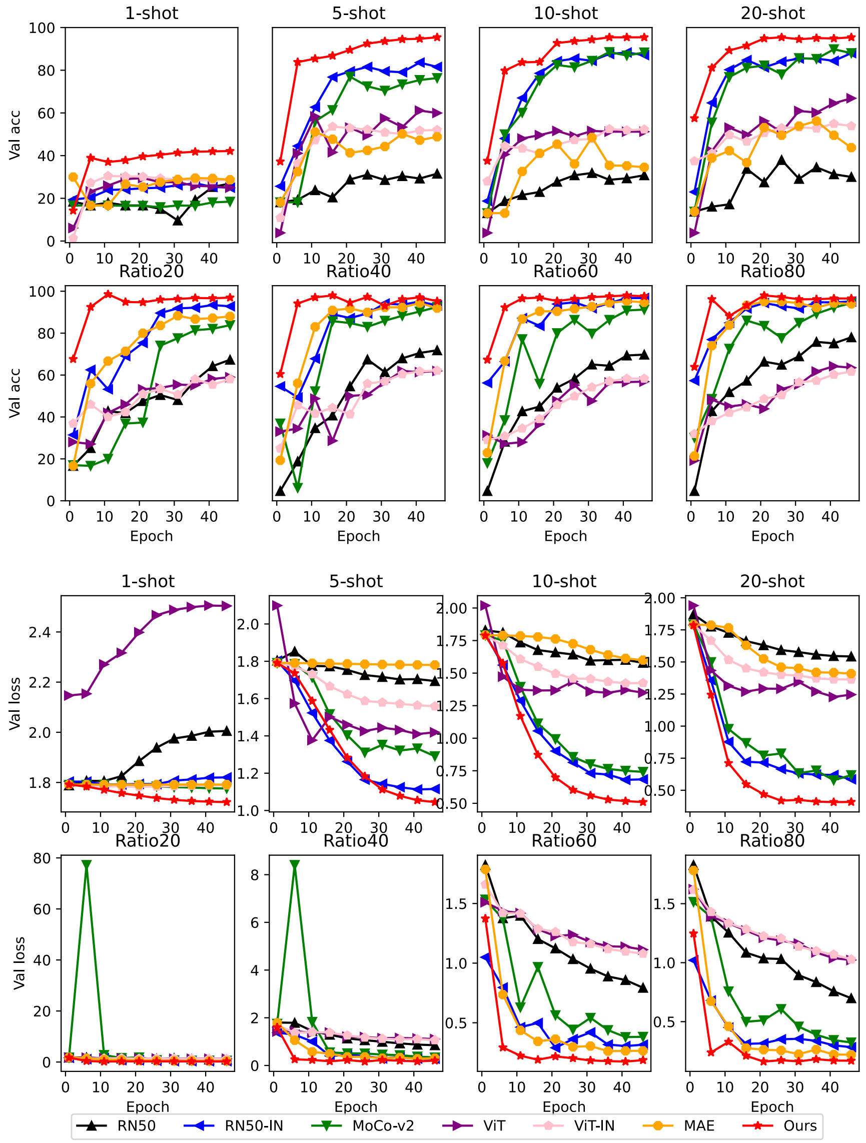

Figure S10. The curves of validation accuracy and loss on IVADLRose.

	1-shot	5-shot	10-shot	20-shot	Ratio20	Ratio40	Ratio60	Ratio80
RN50	24.8	26.4	33.2	39.6	32.8	70.7	76.1	70.7
RN50-IN	25.0	62.7	83.0	84.5	30.2	96.6	91.5	94.8
MoCo-v2	18.4	60.1	84.1	84.8	25.0	95.7	90.6	79.3
ViT	27.6	44.9	48.9	59.5	44.8	72.4	63.2	74.1
ViT-IN	31.4	40.2	47.9	51.1	38.8	74.1	78.6	79.3
MAE	30.0	36.1	47.3	57.4	35.3	61.2	66.7	86.2
Ours	39.9	72.0	86.9	90.1	91.4	97.4	90.6	89.7

Table S11. The testing accuracy of different training methods on CitrusLeaf.

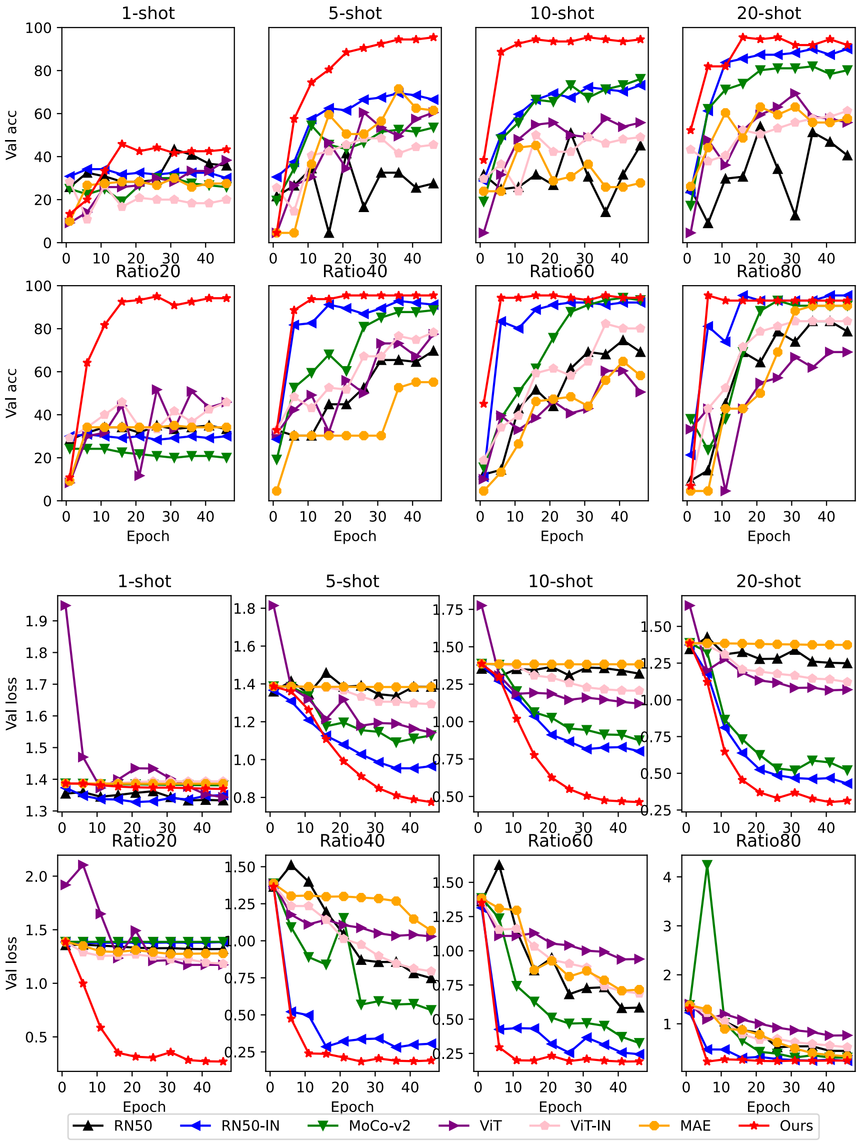

Figure S11. The curves of validation accuracy and loss on CitrusLeaf.

	1-shot	5-shot	10-shot	20-shot	Ratio20	Ratio40	Ratio60	Ratio80
RN50	35.9	35.9	42.7	57.3	57.2	63.6	61.5	70.9
RN50-IN	25.6	62.4	77.8	84.6	84.4	80.3	85.6	84.9
MoCo-v2	30.8	53.0	74.4	83.8	72.3	74.0	82.2	83.7
ViT	33.3	50.4	58.1	64.1	57.2	63.0	66.7	67.4
ViT-IN	29.1	47.9	47.0	60.7	56.1	61.8	67.2	64.0
MAE	23.9	60.7	41.9	59.8	59.0	82.7	84.5	81.4
Ours	46.2	88.9	89.7	85.5	90.8	91.3	92.5	89.5

Table S12. The testing accuracy of different training methods on CGIARWheat.

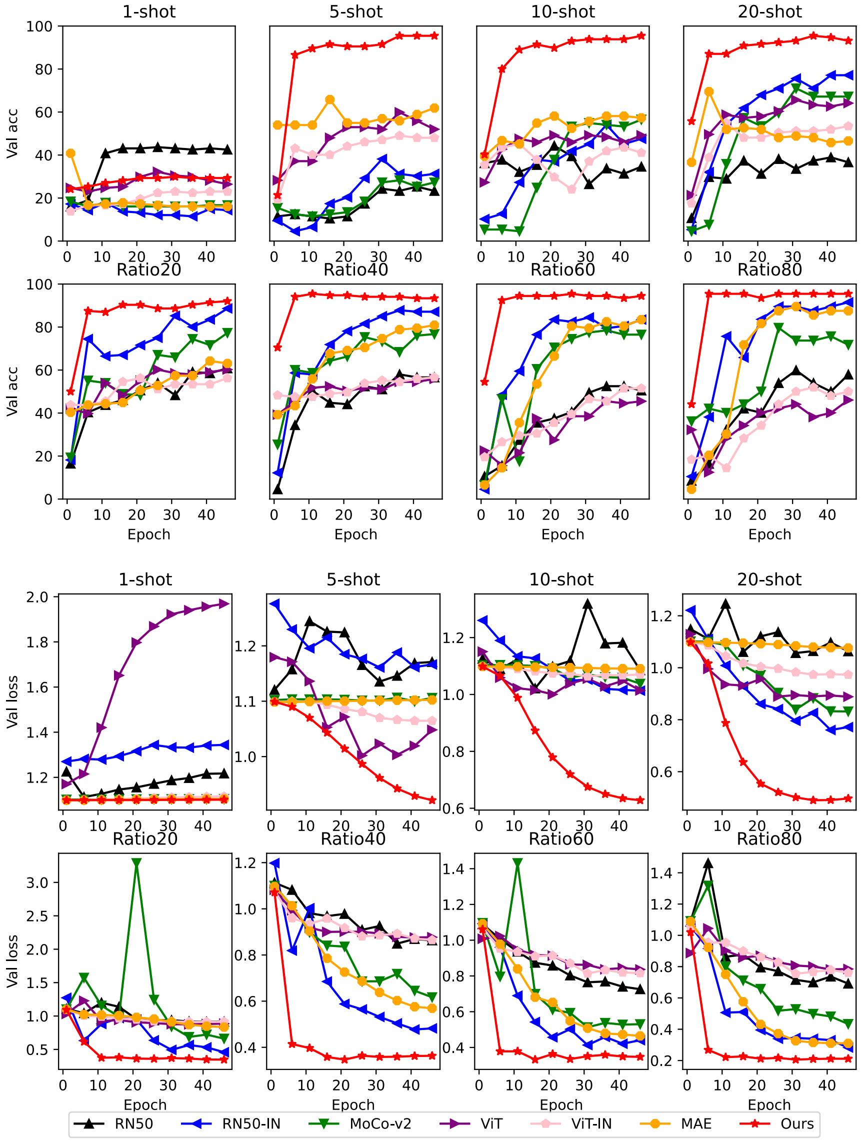

Figure S12. The curves of validation accuracy and loss on CGIARWheat.

	1-shot	5-shot	10-shot	20-shot	Ratio20	Ratio40	Ratio60	Ratio80
RN50	26.1	33.3	38.7	52.3	30.8	78.5	71.6	86.8
RN50-IN	69.4	90.1	100.0	93.7	24.3	97.2	98.2	98.1
MoCo-v2	69.4	87.4	97.3	95.5	41.1	96.3	94.5	96.2
ViT	50.5	56.8	64.9	74.8	70.1	76.6	87.2	94.3
ViT-IN	42.3	48.6	63.1	63.1	67.3	76.6	87.2	96.2
MAE	26.1	61.3	87.4	60.4	74.8	96.3	98.2	98.1
Ours	90.1	96.4	96.4	96.4	100.0	99.1	98.2	100.0

Table S13. The testing accuracy of different training methods on Strawberry2021.

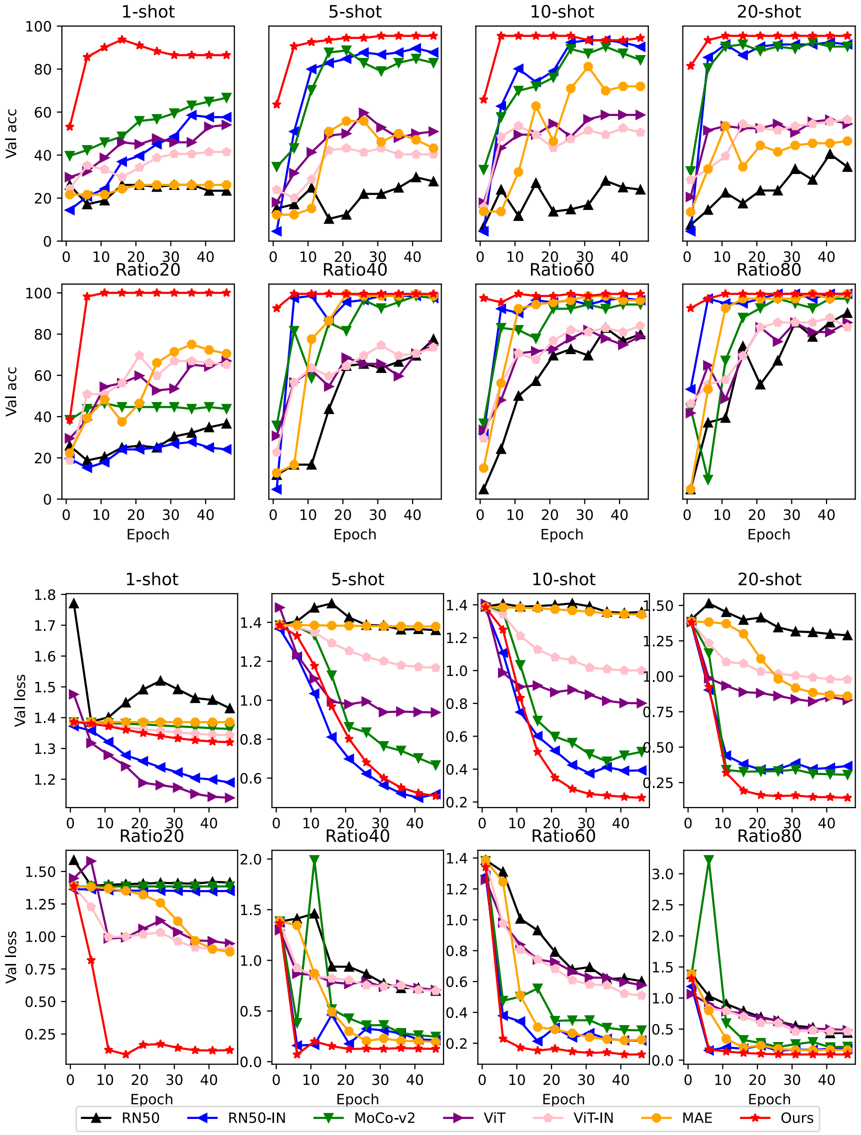

Figure S13. The curves of validation accuracy and loss on Strawberry2021.

	1-shot	5-shot	10-shot	20-shot	Ratio20	Ratio40	Ratio60	Ratio80
RN50	14.9	10.2	14.2	19.6	49.0	59.3	62.2	69.7
RN50-IN	21.7	61.8	75.8	80.6	97.4	98.8	98.5	99.0
MoCo-v2	21.9	53.1	72.0	76.6	92.7	96.6	97.9	98.8
ViT	14.9	23.0	23.7	28.1	42.4	54.7	63.6	67.5
ViT-IN	12.1	18.1	23.1	27.4	42.8	60.0	63.8	68.5
MAE	8.8	21.6	31.6	58.0	95.6	99.3	98.5	99.4
Ours	57.7	98.8	98.7	99.3	99.6	99.6	99.4	99.4

Table S14. The testing accuracy of different training methods on CottonWeedID15.

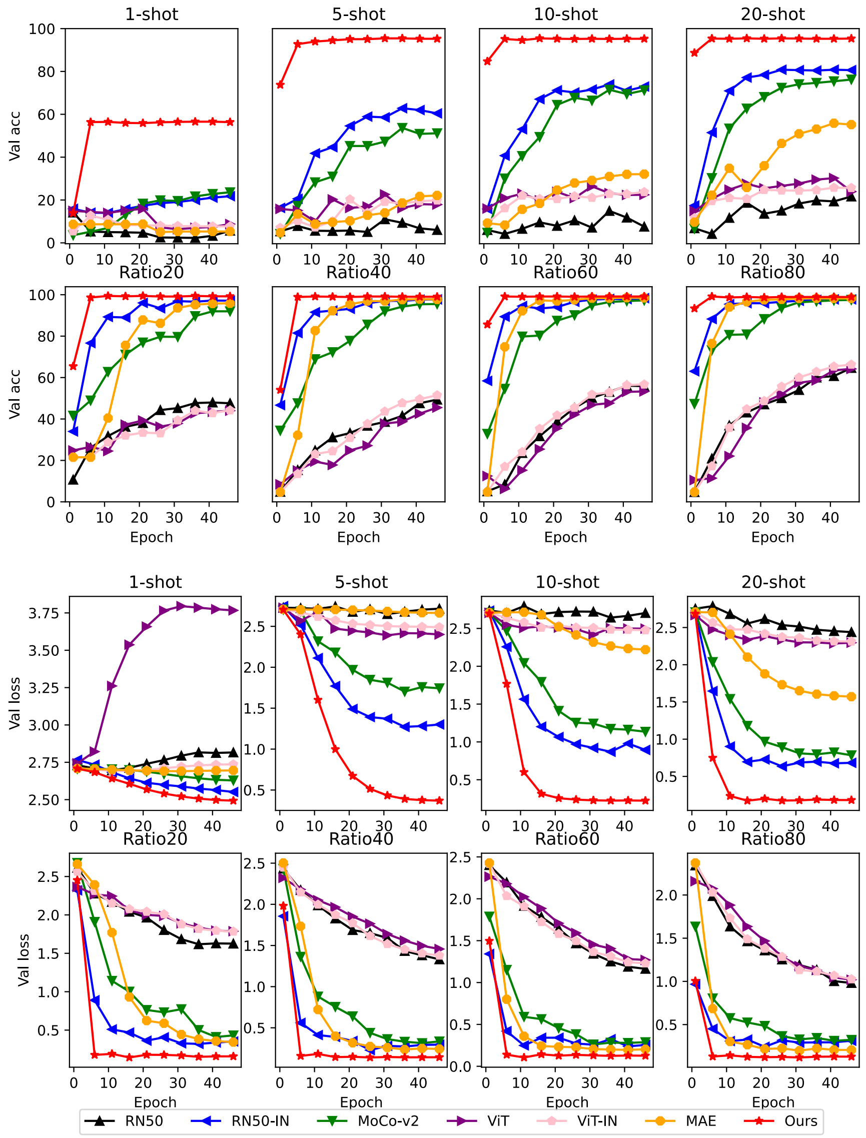

Figure S14. The curves of validation accuracy and loss on CottonWeedID15.
